# Participatory Approaches in the Context of Research Into Workplace Health Promotion to Improve Physical Activity Levels and Reduce Sedentary Behavior Among Office-Based Workers: Scoping Review

**DOI:** 10.2196/50195

**Published:** 2024-06-19

**Authors:** Aidan John Buffey, Christina Kate Langley, Brian P Carson, Alan E Donnelly, Jon Salsberg

**Affiliations:** 1 Department of Physical Education and Sport Sciences Faculty of Education and Health Sciences University of Limerick Limerick Ireland; 2 Physical Activity for Health Research Cluster Health Research Institute University of Limerick Limerick Ireland; 3 University Academy 92 (Old Trafford Campus) Manchester United Kingdom; 4 Public and Patient Involvement Research Unit School of Medicine University of Limerick Limerick Ireland; 5 Public and Patient Involvement Research Unit Health Research Institute University of Limerick Limerick Ireland

**Keywords:** participatory research approach, workplace health promotion, physical activity, sedentary behavior, end user involvement, office based, desk based, intervention, cocreation, public and patient involvement

## Abstract

**Background:**

Participatory research (PR) involves engaging in cocreation with end users and relevant stakeholders throughout the research process, aiming to distribute power equitably between the end users and research team. Engagement and adherence in previous workplace health promotion (WHP) studies have been shown to be lacking. By implementing a PR approach, the insights of end users and stakeholders are sought in the co-design of feasible and acceptable intervention strategies, thereby increasing the relevance of the research.

**Objective:**

This scoping review aims to explore, identify, and map PR techniques and their impact when used in office-based WHP interventions designed to improve physical activity (PA) or reduce sedentary behavior (SB).

**Methods:**

The reporting of this scoping review followed the PRISMA-ScR (Preferred Reporting Items for Systematic reviews and Meta-Analyses extension for Scoping Reviews). A systematic literature search of 5 electronic databases—Web of Science, PubMed, Scopus, Google Scholar, and OpenGrey—was conducted, searching from January 1, 1995, to February 8, 2023. In total, 2 independent reviewers first screened the retrieved articles by title and abstract, and then assessed the full texts based on the inclusion and exclusion criteria. The search strategy and eligibility criteria were developed and guided by an a priori population (office-based working adults), intervention (a PA WHP intervention that took a PR approach), comparison (no comparison required), and outcome (PA or SB) framework. Data were charted and discussed via a narrative synthesis, and a thematic analysis was conducted. The included studies were evaluated regarding the degree of end user engagement throughout the research process and power shared by the researchers, using Arnstein’s ladder of citizen participation.

**Results:**

The search retrieved 376 records, of which 8 (2.1%) met the inclusion criteria. Four key strategies were identified: (1) end user focus groups, (2) management involvement, (3) researcher facilitators, and (4) workplace champions. The degree of engagement and power shared was relatively low, with 25% (2/8) of the studies determined to be nonparticipation studies, 25% (2/8) determined to be tokenistic, and 50% (4/8) determined to provide citizen power.

**Conclusions:**

This review provides a foundation of evidence on the current practices when taking a PR approach, highlighting that previous office-based PA WHP studies have been largely tokenistic or nonparticipative, and identified that the end user is only engaged with in the conception and implementation of the WHP studies. However, a positive improvement in PA and reduction in SB were observed in the included studies, which were largely attributed to implementing a PR approach and including the end user in the design of the WHP intervention. Future studies should aim to collaborate with workplaces, building capacity and empowering the workforce by providing citizen control and letting the end users “own” the research for a sustainable WHP intervention.

**International Registered Report Identifier (IRRID):**

RR2-10.1136/bmjopen-2021-054402

## Introduction

### Background

A growing body of literature in occupational health research, and specifically research into individuals with office- and desk-based occupations, has begun to focus on prolonged occupational sitting [1]. The workplace environment and increased use of computers have been associated with a significant reduction in physical activity (PA) and an increased prevalence of prolonged sitting, especially in desk-based office workplaces [2,3]. Sitting is classified as a sedentary behavior (SB), which is a term used to define any waking behavior with an energy expenditure of ≤1.5 metabolic equivalents of task when sitting, reclined, or in a lying posture [4]. The workplace environment and organizational culture can often facilitate and promote prolonged SB [2,5]. Emerging evidence from 2 previous studies that measured sedentary time via accelerometers indicated that office workers were sedentary for a mean of 75.8% [6] to 81.8% (438.8, SD 51.5 min) of their working hours [7].

Previous research has attempted to reduce or interrupt prolonged occupational sitting in the workplace environment to varying degrees of success, by increasing PA. However, these previous workplace interventions or workplace health promotion (WHP) studies can often be characterized as “one size fits all” interventions, which have taken a traditional top-down, research-driven approach where the end users are considered passive subjects [8-10]. In this review, we define an end user as an individual working in a desk-based office environment or communities such as the organization’s workforce as this population is the target (participants) in WHP studies and the population of interest in this scoping review. Furthermore, this scoping review acknowledged the involvement of relevant stakeholders, following the description provided by Leask et al [11] regarding stakeholders as individuals or groups that are interested or involved in the implementation of an intervention but not the direct end users. Example stakeholders in this scoping review context, but not an exhaustive list, may be family of desk-based office employees, employees not in administrative or desk-based roles within the company, office managers, and company owners. While all these stakeholders are not the specific end users, they have lived experience and knowledge of the workplace and the end users and the ability to inform the design of a relevant, feasible, and acceptable WHP intervention from a stakeholder perspective and facilitate or support changes in the workplace culture, practices, and policy.

To shift research away from top-down “one size fits all” interventions, researchers have begun to take a participatory research (PR) approach to conducting studies. PR incorporates the knowledge and expertise of the end users and relevant stakeholders, thereby increasing the relevance of the research [6,12], and was described by Jagosh et al [10] as the coconstruction of research among people affected by the issues under study and researchers, stakeholders, and decision makers who have the capacity to apply the research findings. Therefore, PR allows for the tailoring of interventions by incorporating the relevant end users and stakeholders within the research process, which has been shown to promote a sense of ownership and aid the acceptability of the research when implemented, if conducted well [6,13]. When a participatory approach is not taken, research has shown that the WHP study may lead to an intervention approach, concept, or format that is inappropriate [14].

Previous research has suggested that the workplace is an ideal and valuable setting for the delivery of preventative health interventions when targeting adults, both healthy and especially those at increased risk of developing chronic diseases [15-17]. Earlier WHP interventions have targeted different aspects of the workplace environment, commuting habits, work schedules or implemented behavior change strategies to increase PA or reduce SB. However, studies targeting behavior change that did not take a PR approach have been shown to be weaker in intervention design [18] as it has been demonstrated to be beneficial for the investigating research team to acquire an understanding of the influences on the targeted behavior in the context in which they occur [19]. For example, in the workplace setting, a manager is an important stakeholder and can provide insights into the acceptability and feasibility of potential intervention strategies [20].

A research priority in WHP interventions is to create a sustainable WHP program after the completion of the study and researcher involvement [21]. Furthermore, maintaining end user adherence throughout the WHP intervention can be difficult, with documented high rates of attrition shown in previous WHP studies [21]. For instance, participants who are highly sedentary before an intervention are likely to return to their previous levels of SB due to increasing work pressures [2]. Including end users and relevant stakeholders with the aim of collaboration, education, and community action can promote active ownership of the research process and sustainability [8,22,23].

PR is a distinctive approach to research and not a particular research method that aims to distribute equitably the power between the research team and the research participants [24]. Therefore, PR is not a research method in and of itself and can take multiple forms and use varying methodologies, methods, processes, and tools [25]. When taking a PR approach, end user and stakeholder involvement can vary in intensity at distinct phases of an intervention (eg, conception, planning, conduct, evaluation, reporting, and dissemination). Conventional research methods such as focus groups and surveys can be adapted and applied in a participatory way, and therefore, any method, tool, or activity can be participatory if chosen or used collaboratively among end users, relevant stakeholders, and their academic partners [25]. The level of end user involvement in selecting, adapting, and implementing a method, process, activity, or tool could be considered more important than the method or technique itself in terms of impact on the WHP intervention. This is supported by Andersson [26], who identified that the quality and impact of the research can increase when end users are involved early in the research process, and reinforced by Vaughn and Jacquez [25], who stated that the level of participation is closely tied to the impact that research will have in real-world settings. The level of participation and power shared between researchers and end users can vary, and when power is not shared between the end user and academic partner in the decision-making process, the research cannot truly be “participatory” [8]. Arnstein [27] states that there is a crucial difference between going through an empty ritual of participation and end users being provided with the real power needed to affect the outcome of the process.

Several systematic reviews and meta-analyses have investigated workplace interventions that were designed to improve PA [21,28-30] or reduce SB within the workplace [31]. These reviews have reported positive overall benefits and that workplace interventions are generally effective in improving PA or reducing SB. However, previous systematic reviews investigating PA WHP studies have stated that the evidence is inconclusive [21,32] and called for more research into the elements of WHP studies that are likely to increase adoption and efficacy within the occupational setting [21]. Previous literature has shown that a PR approach can increase efficacy and lead to successful implementation and greater adherence in health promotion studies [13,18,22]. Thus, this scoping review provides evidence on the use of PR in WHP studies that may lead to greater adoption of PR and success of WHP interventions.

### Rationale

To the authors’ knowledge, the use of PR in WHP studies has not been synthesized, and by examining how PR is currently incorporated within WHP research, we can identify the current available evidence, key approaches and methods, and the scope of reported impacts of PR, thereby providing an overview and identifying key characteristics of the current research that has used PR in WHP interventions aimed at increasing PA and reducing SB.

### Objectives

This study had the following objectives: (1) to identify and map previous literature in which office-based adults have been involved in PR studies and how their involvement shaped the design of the WHP intervention, (2) to identify and discuss the methods implemented in the PR WHP studies, and (3) to discuss the evaluation and outcomes measured in the PR WHP studies included in the scoping review.

### Research Question

How have previous PA WHP studies investigating office-based workers incorporated PR and the end user in their studies and to what reported benefit or detriment?

## Methods

### Protocol and Registration

An a priori protocol was published with *BMJ Open* and is available for this scoping review [33].

The reporting of this scoping review followed the PRISMA-ScR (Preferred Reporting Items for Systematic Reviews and Meta-Analyses extension for Scoping Reviews). The PRISMA-ScR consists of a 22-item checklist [34]. The completed checklist for this scoping review can be found in Table S1 in Multimedia Appendix 1 [16,18,35-40].

This scoping review followed the guidelines and framework published previously by Levac et al [41], who expanded and developed the methodology for scoping reviews by Arksey and O’Malley [42], as planned in the published protocol [33]. This scoping review also followed the more recent methodological guidance published by Peters et al [43,44] for conducting and reporting scoping reviews.

### Eligibility Criteria

In the planning of this scoping review, the research team a priori developed a population, intervention, comparison, and outcome (PICO) framework to assist in the development of the search strategy and the inclusion and exclusion criteria. The PICO framework, as previously published in the scoping review protocol [33], was as follows: the population was office-based working adults; the intervention was PA WHP interventions that used a PR approach; for comparison, we did not wish to compare interventions or treatments (this is typical in some PICO analysis frameworks, where a comparison is not always present); and the outcome was PA or SB levels.

Articles were screened for eligibility related to our inclusion and exclusion criteria (Textbox 1), excluding non–English-language articles.

**Characteristics such as population, language, years considered, focus of the retrieved studies, and publication status; the relevant inclusion and exclusion criteria for each characteristic; and the associated rationale.**

**Inclusion criteria**

- Population: working adults in office environments
- Language: English
- Years considered: January 1, 1995, to February 8, 2023
- Study focus: articles investigating workplace health promotion (WHP) in office-based workplaces that implemented participatory health research techniques that including a physical activity (PA) aspect to the intervention study, for example, increasing PA or decreasing sedentary behavior (SB) using steps or walking, breaks in sitting, exercise, and yoga.
- Publication status: published peer-reviewed journal articles and relevant gray literature, which was defined within this scoping review as theses or dissertations, conference papers, research and government reports, ongoing research, editorials, and textbooks.

**Exclusion criteria**

- Population: home-based “office” workers
- Language: studies written in a language other than English
- Years considered: studies published before January 1, 1995, or after February 8, 2023
- Study focus: studies conducted within the workplace with the aim of improving health; studies based in the community or home and not in the office environment; and health promotion (HP) interventions measuring and targeting psychological or work performance improvements and not measuring or reporting on PA or SB.
- Publication status: any other literature that was not listed in the inclusion criteria, such as websites

**Rationale**

- Population: the focus of this scoping review was to investigate participatory research (PR) in WHP studies in office-based participants and workplaces. Children, teenagers, and retired adults would not fit our eligibility criteria of “working adults.” Non–office workers and home-based workers may have different “health” needs related to the working environment.
- Language: the reviewers only speak English, and feasibility considerations (eg, limited resources) prevented the use of translation services.
- Years considered: a wide period was established to capture all relevant WHP research. The years considered were cut off at 1995 as this is when guidance was published by Green et al [45] for the development of PR in HP and, therefore, implemented into research practices following this year.
- Study focus: the focus of the overall research question of this scoping review was specific to PR in WHP research in office-based workplaces. Other work-based environments may carry different health-associated risks, priorities, or safety concerns, which would not be comparable to those of an office-based environment. Including PA or SB as outcome measures would allow for an evaluation of the included studies and a discussion on the effectiveness of taking a PR approach in those WHP studies. We excluded studies that did not measure or reported PA or SB and included those that did to address our research question as these outcome variables were the primary outcome variables of interest. Of the included studies, those that reported further outcome variables such as psychological well-being, diet, or work performance were eligible and included; however, we did not report or discuss these additional outcome variables in this scoping review as they were outside the scope.
- Publication status: the aim of this scoping review was to capture a wide range of literature, so including gray literature ensured a more complete search and minimized publication bias.

### Data Sources, Searches, and Study Selection

A total of 5 electronic databases was systematically searched by 2 independent reviewers (AJB and CKL). These databases were Web of Science, PubMed, Scopus, Google Scholar, and OpenGrey. The full electronic search strategy for each database was previously published with the protocol [33].

The first search was executed on January 17, 2022, retrieving articles between this date and January 1, 1995. This criterion was used for all the included databases. These years were considered as part of our inclusion and exclusion criteria (Textbox 1). The second search to ensure all recent and relevant literature was retrieved was executed on February 8, 2023, retrieving articles between this date and January 17, 2022.

The retrieved articles were exported from the 5 electronic databases to EndNote (Clarivate Analytics), where duplicates were removed via the EndNote function and manually when a duplicate was missed by the software.

**Figure 1:**
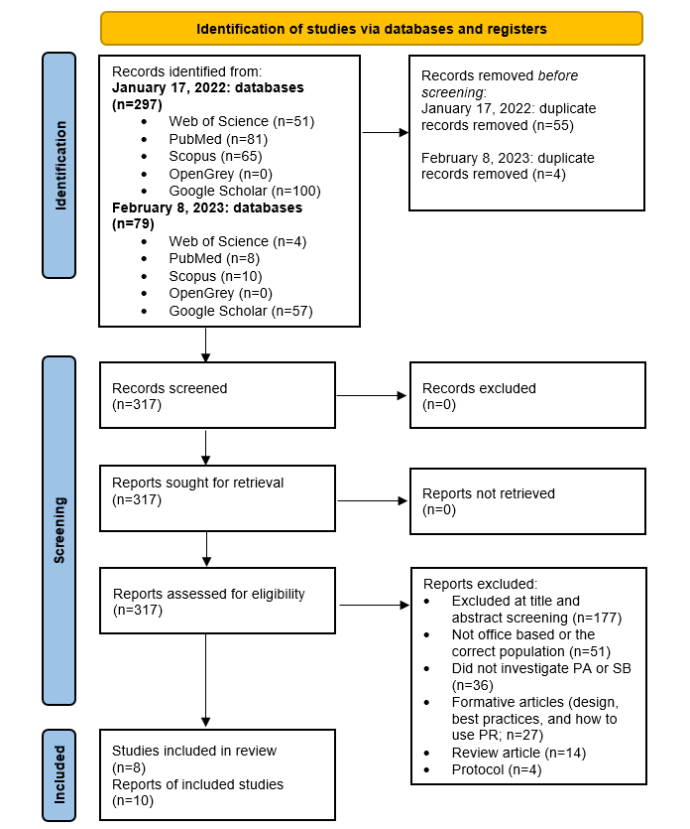
PRISMA (Preferred Reporting Items for Systematic Reviews and Meta-Analyses) flow diagram illustrating the number of studies retrieved and the screening process with reasons for exclusion, leaving N=8 included studies. The first date (January 17, 2022) refers to the first search completed, and the second date (February 8, 2023) refers to the second search. Further information can be found in the Study Selection section. PA: physical activity; PR: participatory research; SB: sedentary behavior.

Following duplicate removal, retrieved articles were reviewed by 2 researchers independently (AJB and CKL); the retrieved articles were screened first by title and abstract based on the inclusion and exclusion criteria and then by full text. Any discrepancy between the 2 reviewers regarding eligibility was discussed until consensus was reached. The PRISMA (Preferred Reporting Items for Systematic Reviews and Meta-Analyses) flow diagram was populated to show the number of articles retrieved, screened, and excluded (with reasons) before reaching our number of included studies (Figure 1).

A comprehensive evaluation of the retrieved articles was conducted, which involved an informal multistep iterative approach and screening system by the lead researcher (AJB) and second reviewer (CKL) assessing whether a retrieved study had taken a PR approach if the article met all other inclusion criteria. The first step or indication that both reviewers would try to identify when screening a retrieved article was the keyword “participatory” or a related synonym; when this keyword (“participatory”) or related synonyms were not present, the reviewers would search for words such as “co-design,” “co-development,” and “co-produced.” If these words were still not present within a retrieved manuscript, a search would be conducted for key terms such as “end user,” “stakeholder,” “manager,” and “volunteer.” Finally, a further screening of the retrieved articles would then be conducted looking for specific nuances or characteristics of PR, such as focus groups, surveys, meetings or community boards, panels, or groups that were conducted or mentioned to be formed in the retrieved article with the objective of designing, tailoring, or facilitating an intervention. If none of the aforementioned key terms or characteristics of PR were present within a retrieved manuscript, the study was excluded.

No in-depth assessment of whether a study was participatory was completed during the screening phase. For example, studies did not need to meet certain requirements to be considered participatory. However, the level of participation was examined during data extraction and when writing the narrative synthesis of all the included studies in this scoping review.

### Data Extraction and Charting Process

Data from the included studies were charted independently by one reviewer (AJB). The second reviewer (CKL) charted 2 randomly selected articles independently to duplicate and confirm the data charting process. It was previously proposed that the second reviewer would independently duplicate 10% of the included articles [29]; due to <10 articles being included, 2 articles were deemed more than the agreed upon 10% and sufficient for confirming the data charting process.

Data were extracted and charted using a Microsoft Excel (Microsoft Corp) sheet table. The Microsoft Excel table had been piloted by the research team and peer reviewed during publication of the scoping review protocol.

### Data Items

When charting the data from the included studies, we sought to retrieve, extract, and chart information on variables such as study design, study purpose and aims, PR approach taken, whether oversight was included and whether this oversight was a participatory group or researcher oversight, level of involvement from end users and stakeholders throughout the study, intervention focus, data collection methods, study outcomes (primarily related to PA and SB), data analysis, and whether the studies self-evaluated the PR techniques they implemented. Further information on each of the data items described has been provided within the published protocol of this scoping review [33]. These data items were the headings used in the data extraction and charting process. Each data item had an associated question that was used to retrieve and chart the extracted information from the included articles.

### Data Synthesis and Analysis

As is a standard approach when conducting scoping reviews [41,42,46], a critical appraisal of the included studies or data was not conducted. This was decided a priori, as documented in the study protocol [33].

### Synthesis of Results

Following data extraction and charting, we summarized our findings via a narrative synthesis, providing a descriptive summary of the included studies and charted data. A qualitative thematic analysis was conducted using Microsoft Excel according to the approach published by Bree and Gallagher [47] and following the guidance of Braun and Clarke [48,49].

The studies were evaluated based on the degree of participation and power shared with end users when making important decisions with the public, end users, and relevant stakeholders from nonparticipation to tokenism to citizen power using the ladder of citizen participation by Arnstein [27], which is an 8-rung ladder. There are 2 rungs (manipulation and therapy) categorized under nonparticipation, 3 rungs (informing, consultation, and placation) that fall within tokenism, and 3 rungs (partnership, delegated power, and citizen control) that form citizen power [27]. Therefore, the ladder by Arnstein [27] allowed the authors to distinguish and compare the level of participation and access to power in the included studies [50]. Each of the 8 different rungs of the ladder relates directly to the extent to which end users have attained decision-making power, with the highest rung signifying complete citizen control [51]. The highest rung of the ladder and the top 3 rungs that fall under citizen power are deemed by Arnstein [27] to represent actual power transferring to the end users [27]. Therefore, when evaluating the included studies, those rated on the lower rungs of “nonparticipation” and “tokenistic” would be deemed to be less participatory than those rated as providing “citizen power,” and it was determined that the highest rungs should be preferred over the lower rungs [27]. The simplicity of the ladder has been critiqued by Arnstein [27] herself and by more recent authors such as Tritter and McCallum [51] regarding the fact that, within the real world, programs and people may in fact have 150 rungs with less sharp and clear distinctions among them and that user engagement and empowerment is a complex phenomenon. Therefore, certain characteristics of PR could be applicable to multiple rungs depending on how they are illustrated, and the dynamic structure and layered complexity of end user involvement may not be captured in the fluctuating power dynamics that are generally present when attempting PR in the real world [51].

However, for the purpose of this review, the ladder by Arnstein [27] provides a useful heuristic to evaluate and determine the level of involvement and shared power provided to the end user in the included studies due to its simplicity and provocative thought-provoking nature when assessing how “participatory” the included studies were. While it was not expected that all the included studies would provide citizen control or that this would be better in all research designs or contexts, the authors anticipated their level of involvement and power shared to fall within the top 4 rungs as the last rung of tokenism does grant some degree of influence in the process but anything below lacks authentic involvement [27].

### Patient and Public Involvement

As we were conscious of the time burden and commitment at this stage, end users and stakeholders were not involved in the development or conduct of this scoping review. However, review findings will be shared with end users and stakeholders involved in a future project, which will take a PR approach, to inform and facilitate an office-based PA WHP study, which will be co-designed, facilitated, implemented, evaluated, and disseminated.

## Results

### Study Selection

The systematic search strategy of 5 electronic databases identified 297 articles upon first search and an additional 79 articles with the updated second search, totaling 376 articles. Following the screening process of all eligible articles, which was conducted independently by 2 reviewers (AJB and CKL), of the 376 studies, 8 (2.1%) met the inclusion criteria (Figure 1). Of the 8 included studies, 2 (25%) provided additional reports, one in the form of a study protocol [52] and the second in the form of a second manuscript reporting findings obtained from the same study [53]; however, these reports were combined and only discussed as the manuscript retrieved and included via the search strategy, and therefore only 8 studies will be referred too.

### Study Characteristics

A total of 38% (3/8) of the included studies were pilot studies [35-37] that did not randomize participants and used a pretest-posttest [35,36] or a posteriori quasi-experimental study design [37], whereas 50% (4/8) were cluster-randomized trials [16,18,38,39]. The final study had a longitudinal pretest-posttest study design [40]. In total, 38% (3/8) of the studies took a mixed methods research approach collecting and analyzing both quantitative and qualitative data in the same study [35-37,40]. The duration of the studies ranged from 3 weeks [36] to 18 months [40].

The sample size ranged from 5 [36] to 585 [16] across the included studies, and they were conducted in various countries, such as the United Kingdom [35,36], Australia [18,37], China [38,39], Sweden [40], and Singapore [16]. One of the included studies sampled women only [16], whereas the rest were mixed-sex studies [18,35-40]. Participants’ occupations varied across the included studies, and they were based in large IT organizations [38], telecommunications organizations (administrative and clerical workers) [37], government organizations (clerical, data entry, and call center office workers) [18], and workplaces in sectors or industries that were primarily office based and sedentary in nature (government administration and finance departments) [16]. Some participants were office workers in desk-based occupations (education or research, administration, human resources, accountancy, sales, and IT) [36]; worked within a department in an academic institution (university) [35]; and were part of a singular organization that serviced a municipality with 56,000 inhabitants and carried out assignments such as running and developing schools, providing social services, and conducting urban planning [40]. The final study did not provide specific occupations but stated that the included worksites were located in the Yangtze River Delta in China and comprised primarily desk-based occupations [39].

Further study characteristics can be found in Table 1, and the intervention components and reported outcome variables can be found in Table S2 in Multimedia Appendix 1.

**Table 1:** Characteristics of the included studies, including the year of publication, country of origin, number of participants, number of end users involved in the participatory research approach, and the inclusion and exclusion criteria.

Study	Year	Design	Country	Participant composition	Inclusion and exclusion criteria
Blake et al [38]	2019	2-arm cluster-randomized waitlist control trial	China	Total: N=282 Intervention: n=196 (96 male participants, 49%; 97 female participants, 49.5%; and 3 not specified, 1.5%) Control: n=86 (46 male participants, 53.5%; 37 female participants, 43%; and 3 not specified, 3.5%) Participatory research: organizational committee=4 (2 team leaders and 2 HR^a^ officers); team leaders=31 (invited by the organizational committee to act as intervention facilitators)	Inclusion criteria: eligible clusters were 2 sites of a large IT organization, and eligible participants were employees of the organization.
Gilson et al [37]	2016	Pilot—a posteriori quasi-experimental design	Australia	Intervention protocol 1 (strategies and no prompts): n=33 (27 male and 6 female participants) Intervention protocol 2 (strategies and prompts): n=24 (19 male and 5 female participants) Participatory research: participatory 1-hour workshop=10-15; aimed to identify occupational strategies for “sitting less and moving more”	Inclusion criteria: all members of each work team were eligible to participate. Exclusion criteria: N/A^b^
Griffiths et al [36]	2022	Pilot—mixed methods intervention	United Kingdom	Pilot: n=5 (2 male and 3 female participants) “Needs analysis” questionnaire: n=157 (19 male and 138 female participants) Participatory research: coproduction development focus group=11 (4 male and 7 female participants); range of employees—management=3; sales=2; IT=3; HR=3 4 fields of employment from 6 different organizations: public health=2; IT=5; energy supplier=3; education=2	Inclusion criteria: (1) adults (aged ≥18 years), (2) occupying seated job roles (defined as sitting for ≥6 hours during work hours), and (3) being physically inactive (defined as not meeting UK PA^c^ guidelines) Exclusion criteria: (1) individuals who currently used active workstations (defined as reporting regular use of sit-stand, treadmill, or pedal desks), (2) inability to complete any desk-based focused PA, (3) failure to occupy sedentary jobs, and (4) meeting and exceeding UK PA guidelines
Kong et al [39]	2022	Group randomized controlled trial	China	Total enrolled: N=955 (4 worksites) Intervention: n=464 (2 worksites) Control: n=491 (2 worksites) Baseline—intervention: n=216 (2 worksites); control: n=172 (2 worksites) Final evaluation—intervention: n=159 (2 worksites); control: n=119 (2 worksites) Intention-to-treat analysis—intervention: n=216 (2 worksites); control: n=172 (2 worksites) Participatory research: an EAB^d^=4 to 7 employees from all occupational sectors in the worksite in each worksite (4 worksites × 4 to 7 = approximately 16 to 28; unknown number of occupational sectors and whether the EABs where present in the control worksites, so may be 2 worksites × 4 to 7 = approximately 8 to 14)	Participant inclusion criteria: (1) ages of ≥18 years, (2) full-time employees, (3) not having received clinical weight-loss treatment, (4) not pregnant at the time of recruitment, and (5) having signed informed consent form Participant exclusion criteria: N/A Worksite inclusion criteria: (1) large proportion (>50%) of desk-based employees, (2) operating for >3 years, and (3) never having hosted a health management program
Mackenzie et al [35]	2015	Pilot—uncontrolled pretest-posttest intervention (mixed methods evaluation)	United Kingdom	Total: N=24 Completers (statistical analysis): n=17 (4 male and 13 female participants) Participatory research: intervention development 1-hour focus group=7/11; 4/11 were unable to attend and submitted suggestions via email	Inclusion criteria: all employees of ScHARR^e^ were eligible to participate in the study. Exclusion criteria: N/A
Parry et al [18]	2013	Parallel-arm cluster-randomized trial	Australia	Total: N=133 (18% male and 82% female participants) Participants with complete data and included in the analysis: n=62 (19% male and 81% female participants) Participatory research: participants from all 3 workplaces or interventions were asked to attend 2 structured meetings at their workplace to discuss and develop their specific workplace intervention.	Inclusion criteria: workers participating in office-bound duties for ≥6 hours per day and working ≥4 days per week Exclusion criteria: participants were only excluded if they were unable to wear an accelerometer due to disability or if they were confined to a wheelchair.
Tan et al [16]	2016	2-arm cluster-randomized trial	Singapore	Total: N=585 (585 female) Intervention: n=287 (287 female); intervention—PA analyzed: n=234 Control: n=298 (298 female); control—PA analyzed: n=196 Participatory research: all participants in the tailored intervention received 3 participatory workshops focused on participatory skill-building activities, peer support, goal-setting exercises, and problem-solving discussions to attain individual goals and overcome individual barriers.	Workplace inclusion criteria: (1) workplaces in sectors or industries that were primarily office based and sedentary in nature (ie, government administration and finance departments), (2) workplaces that were able to recruit at least 30 female employees engaged in desk-based jobs (sitting ≥50% of working hours), and (3) agreement to permit up to 10 hours of paid work time during the course of the study (12 months) for the recruited employees to participate in pretest-posttest data collection and intervention activities Participant inclusion criteria: (1) being female, (2) being aged 25 to 49 years, and (3) having a sedentary job (at least 50% of work hours seated) Participant exclusion criteria: (1) being pregnant or lactating, (2) diagnosis of osteoporosis, (3) diagnosis of kidney problems, and (4) participation in another health program that addressed diet or PA
Wahlström et al [40]	2019	Longitudinal mixed methods	Sweden	Total (baseline characteristics): N=152 (50 male and 102 female participants) Interviews: n=70 (17 male and 53 female participants) Focus groups: n=15; flex office [activity-based work office where there are no fixed workstations, but instead various spaces in the office, which are designed to support the performance of different work tasks]: n=43; cell office [the most common office types are cell offices and open landscapes with fixed workstations]: n=43 From within the organization, 7 female employees volunteered to be health promoters or inspirers. Managers were involved along with a health strategist from within the organization and the organization’s communication department; however, the PA-promoting program was initiated by the researchers with collaboration between researchers and workplace representatives, but the exact number was not stated.	Inclusion criteria: (1) aged 18 to 63 years, (2) working ≥75, (3) >60% of work hours inside the office, and (5) not planning to retire or relocate to another worksite during the study period Exclusion criteria: N/A

^a^HR: human resources.

^b^N/A: not applicable.

^c^PA: physical activity.

^d^EAB: employee advisory board.

^e^ScHARR: Sheffield Centre for Health and Related Research.

### Synthesis of Results

#### Overview

In this section, we provide a narrative synthesis that focuses on how participants, end users, and relevant stakeholders of the included studies were involved in the WHP studies and how their involvement shaped the design of the WHP intervention. This narrative synthesis was guided by a thematic analysis that identified themes related to how end users, stakeholders, and researchers were involved in the research process when taking a PR approach and what role they played (Table 2). Further information on the PR methods implemented in the included studies, including how they engaged end users and relevant stakeholders, the number and duration of any meetings and activities, how many end users and stakeholders were involved in the PR method or methods, and the content and agenda of any meetings or activities when engaging with the end users and relevant stakeholders, can be found in Table S2 in Multimedia Appendix 1.

**Table 2:** Components of the thematic analysis of end user, stakeholder, and researcher involvement in the participatory research process and overall conduct of the study. The table highlights primary themes and subthemes and provides a description of each theme with supporting illustrative quotes with references.

Themes, subthemes, and descriptions			Illustrative quotes and references
**Theme 1: participant workshops, focus groups, meetings, or brainstorming sessions**			
	This theme describes the format of how the participants were typically asked to express their opinions, views, and personal experiences in relation to designing or evaluating a WHP^a^ intervention.		—^b^
	**Subtheme 1.1: researcher-provided evidence**		
		This subtheme describes how, as part of meetings with participants, end users, and stakeholders, the research team would provide evidence that supported what they wished to codevelop with the participants, which was a WHP intervention designed to increase PA^c^ and reduce SB^d^.	“During the workshop, researchers reviewed evidence on the benefits of reducing sitting and increasing physical activity...” [37] “In the first focus group, participants were educated via an online presentation on the importance of breaking up sitting time regularly for cardiovascular health...” [36] “...this involved an initial description of the associations between prolonged sitting and health.” [35]
	**Subtheme 1.2: workers identified strategies for intervention development or promotion**		
		This subtheme describes how participants and workers were asked to contribute and discuss their ideas and strategies for the development, implementation, and promotion of a WHP intervention at their workplace.	“...asked to comment and discuss the concept of breaking up sitting time and the initial perceived challenges.” [36] “...workers identified and discussed occupational strategies for ‘sitting less and moving more.’” [37] “...were asked to comment on and discuss how they would prefer to break up sitting time.” [36] “...‘brainstorming’ session where strategies were identified by participants on how to reduce workplace sitting time.” [35] “Eleven staff from (ScHARR) volunteered to be a part of an intervention development focus group...participants who were unable to attend the meeting submitted suggestions via email.” [35] “Participants from all 3 interventions were asked to attend two structured meetings at their workplace to discuss and develop interventions...to develop workplace specific interventions as part of the participatory approach.” [18] “During the first meeting participants ‘brainstormed’ options to promote their specific intervention (active office, physical activity, or office ergonomics)...Between meetings participants were encouraged to think about specific strategies...At the second meeting, 2-3 weeks following the first meeting, participants shared their ideas and rated potential strategies in terms of feasibility and effectiveness.” [18] “An employee advisory board, which consisted of four to seven employees from all occupational sectors in the work site, was established in each work site and worked closely with the research team to design and implement intervention activities.” [39]
	**Subtheme 1.3: workplace centered**		
		This subtheme describes how meetings and workshops took place at the workplace rather than asking participants to come to the research team.	“...workers attended a one-hour workshop (n = 10-15) held at the work site.” [37] “Participants from all 3 interventions were asked to attend two structured meetings at their workplace to discuss and develop interventions.” [18]
	**Theme 2: manager or management**		
		This theme describes how managers or management of the workplace acted as gatekeepers and were often used or asked to contribute in different formats or circumstances compared to workers.	—
	**Subtheme 2.1: management creating study materials**		
		This subtheme describes how management took an active participation in creating study materials, thereby bringing more relevance and familiarity to the developed posters and videos.	“Management were involved in the design and development of promotional posters and exercise videos.” [38]
	**Subtheme 2.2: management distributing study materials**		
		This subtheme describes how the managers acted as gatekeepers to the workplace and were used to pass information or study materials from the research team to the participants and workplace.	“Managers of the teams distributed emails to their administrative and clerical workers.” [37] “Managers distributed questionnaires...” [40] “Information to managers were communicated via manager meetings and e-mails, and they were asked to disseminate and discuss the messages from the campaigns and workplace meetings.” [40]
	**Subtheme 2.3: management leading in data collection and setting up intervention components**		
		This subtheme describes how individuals with managerial roles assisted with data collection and study design and provided support throughout the study.	“Management provided good technical support throughout the study period, including setting up the development of the pop-up window system and online exercise log recording system.” [38] “The two sites were then randomly allocated to intervention/control groups by a senior manager who was not part of the research team.” [38]
	**Subtheme 2.4: invitation from the management or workplace**		
		This subtheme describes how invitations to participate in the development of the WHP study were sent via the workplace itself rather than through the research team.	“The HR department invited all employees from both sites to participate in the study via their company intranet webpage.” [38]
**Theme 3: researcher facilitator**			
	This theme describes how the research team or lead research facilitator would communicate with team leaders or managers to help with the facilitation of the WHP intervention.		“At this meeting (second meeting) an action plan was developed, and the facilitator communicated with team leaders and management to help implementation.” [18] “Information to mangers were communicated via manager meetings and emails.” [40]
**Theme 4: workplace champions, health inspirers, team leaders, and health strategists**			
	This theme describes how the studies recruited participants from the workplace for elevated positions within the study to lead by example or provide more in-depth insights when codeveloping components of the WHP study.		“Individuals within the organisation became ‘health inspirers’ and assisted with the development of the communication campaigns.” [40] “In the workshops, where communication campaigns were developed, 2-7 ‘health inspirers’ participated, which were fewer than expected.” [40]
	**Subtheme 4.1: involved in intervention or implementation delivery or provision of support in increasing PA**		
		This subtheme describes how participants, managers, workplace champions, health inspirers, team leaders, or health strategists, and stakeholders were involved in the delivery of the intervention and supported WHP and increasing participants’ PA.	“...co-workers were involved in provision of support for physical activity.” [38] “...participatory approach to intervention delivery.” [38] “Organisations communication department and internal health strategist delivered all material via posters, table-tops in meeting rooms and break out spaces, as well as posts on the workplace intranet.” [40] “At this meeting (second meeting) an action plan was developed, and the facilitator communicated with team leaders and management to help implementation.” [18] “Management provided good technical support throughout the study period, including setting up the development of the pop-up window system and online exercise log recording system.” [38]

^a^WHP: workplace health promotion.

^b^Not applicable.

^c^PA: physical activity.

^d^SB: sedentary behavior.

To evaluate the level of involvement and power shared with the end users and relevant stakeholders in the WHP studies, we used the ladder of citizen participation by Arnstein [27].

#### Level of Participation (Ladder of Citizen Participation)

In this scoping review, we aimed to evaluate the role and level of participation that the end users and stakeholders had in three aspects of the included studies: (1) the conception of the WHP study and intervention, (2) the data collection, and (3) the analysis and reporting of the included studies, as documented a priori in our published protocol [33].

##### Conception

When evaluating the included studies and the degree of participation and power shared with their participants within the conception and design of the WHP studies, 25% (2/8) of the studies were deemed as nonparticipation [38,40]. The studies by Blake et al [38] and Wahlström et al [40] were deemed to be nonparticipation as they stated the following:

The IT organisation was not involved in the study conception or design and had not financially supported the research or research team.

38

In parallel with the relocation, a PA-promoting program initiated by the researchers was developed and implemented.

40

A total of 25% (2/8) of the included studies [16,37] were determined to be tokenistic in how they involved the participants and relevant stakeholders when conceiving and designing their WHP interventions. Gilson et al [37] described that, as researchers, they reached a consensus from the ideas generated by the workers regarding the design and number of strategies in the WHP study. While Tan et al [16] held workshops and participants self-selected their own intervention activities, the individually tailored strategies still needed to meet the prescribed 5 to 10 minutes of exercise breaks as designated by the research team. This level of involvement of the end users could be seen as “consultation” or “placation” on the ladder of citizen participation by Arnstein [27] as the researchers retained the right to judge the end users’ ideas and advice.

In total, 50% (4/8) of the studies were evaluated and determined to have provided citizen power, where the participants discussed and developed the WHP intervention and the planning and decision-making were shared between the researchers and the participants [18,35,36,39]. While the level of participation in 75% (3/4) of these studies was evaluated as “citizen power,” their level of participation was found to be at the lowest rung under “citizen power” of the ladder by Arnstein [27], “partnership.” They were determined to be at the “partnership” rung as the researcher facilitated the discussions and communicated with team leaders and management concerning the implementation of the WHP study [18]. Kong et al [39] described the intervention as a community-based participation intervention where community (worksite) employees and researchers engaged as equals in a cooperative process; however, the power shared was not clearly documented, and the statement was merely cited as what a community-based participation intervention is, referencing Minkler and Wallerstein [54]. Kong et al [39] did state that they formed employee advisory boards (consisting of 4 to 7 employees), which were established in each worksite from all occupational sectors to “...work closely with the research team to design and implement intervention activities,” whereas Griffiths et al [36] described a “...compromise between the stakeholder input and researchers’ evidence-focused approach” and, furthermore, that they “...were able to include the participants views and opinions within the intervention design.” Finally, Mackenzie et al [35] held a brainstorming session with participants, who identified the strategies to reduce workplace sitting in the WHP study.

##### Data Collection and Data Analysis and Reporting

When originally planning and proposing our evaluation of the included studies, we had stated that we would evaluate whether participants and stakeholders were involved in data collection and the data analysis and reporting of the study. Upon extracting and charting the data from the included studies, we found that none of the studies included participants or stakeholders within the data collection, analysis, or reporting process. However, participants were included and took part in facilitating and implementing the intervention, and therefore, we retrospectively included a narrative synthesis on how participants were involved in the facilitation of the WHP study.

##### Facilitation of the Included WHP Studies

The larger components of the WHP interventions can be found in Table S3 in Multimedia Appendix 1. In this section, we provide a narrative synthesis on how end users and stakeholders assisted in facilitating the WHP studies. In this scoping review, the authors use the term “facilitation” to describe methods and processes that are active components of implementation, where individuals, end users, and stakeholders who are defined as facilitators enable and influence the implementation process [55].

One study provided no indication or description of end users assisting with the facilitation [36]. In total, 38% (3/8) of the studies enlisted the workplaces’ assistance in recruiting participants to the study [16,37,38]. Tan et al [16] enlisted their workplace coordinators to facilitate within-cluster recruitment to the study. The workplace coordinators were provided with resources by the research team to aid this recruitment, whereas managers were directly involved and facilitated recruitment by distributing recruitment emails to their administrative and clerical workers in the workplace study conducted by Gilson et al [37]. Management involvement was evident in the remaining 50% (4/8) of the WHP studies [18,35,38,40]. This management involvement ranged from emails [35] to introduce, support, and carry forward the intervention [35] to distributing questionnaires supporting and inspiring a healthy lifestyle, and management was asked to discuss and disseminate messages from the WHP campaigns at workplace meetings [40]. Blake et al [38] described the most involvement from managers, who delivered 30-minute orientations to team leaders on the WHP intervention to demonstrate senior management commitment, with the senior manager randomizing the 2 sites and being involved in the design and development of promotional posters and exercise videos. The management team also provided technical support, including the development and installation of a pop-up window system and web-based exercise log recording system [38]. Though not directly management involvement, the health resources department was responsible for inviting all employees from the worksite to participate in the study via their company intranet web page [38]. Whereas management involvement was not clearly described by Parry et al [18], other than “helped with implementation”.

The most frequent component of facilitation was the empowerment of employees in roles such as workplace champions. This term differed among the studies, with other titles used such as “team leaders” [16,38], and “health inspirers” [18]. Blake et al [38] asked their workplace champions to lead group exercise classes at allocated times, and the champions were involved in the provision of support for PA. This was similarly asked of the workplace champions in the WHP study by Mackenzie et al [35], who asked workplace champions to promote standing and walking meetings or sessions as well as incidental walking (talking and not emailing) and support lunchtime walks. This was further evident in the study conducted by Wahlström et al [40], who asked the health inspirers to assist with the development of the communication campaign and support and inspire coworkers in attaining a healthy lifestyle. Unfortunately, it was not clear to what extent workplace champions facilitated the study carried out by Parry et al [18] other than that they “helped implementation,” whereas the study by Kong et al [39] was the only one to form an employee advisory board that worked closely to design and implement intervention activities with the research team. However, the extent of involvement of the end users, management, or relevant stakeholders in the implementation of all the intervention activities compared to the research team was not clearly indicated in the study [39], but it was stated that the employees voluntarily set up several exercise teams that monitored and attempted to improve their daily behaviors by recording their daily exercise and reminding each other to exercise daily. These exercise teams would meet regularly to exercise at an agreed time and place together [39].

The final forms of facilitation that we observed in the included studies had to do with the marketing of the interventions. Blake et al [38] formed an organizational committee made up of 2 team leaders and 2 human resources officers, who developed and implemented company policy on the internal marketing of the intervention. In the study by Wahlström et al [40], the organization’s communication department and the internal health strategist distributed all materials via posters, tabletops in meetings, and breakout spaces, along with posts on the workplace intranet.

#### Impact on PA and SB Levels

Of the 8 included studies, 1 (12%) [35] did not report PA as an outcome variable, but the remaining 7 (88%) studies did so in varying formats and across different intensities. Overall, 86% (6/7) of the studies did report an increase in PA, whereas Griffiths et al [36], who only measured and reported moderate to vigorous PA, found no change in their 2-week pilot study with 5 participants.

When investigating SB, 62% (5/8) of the included studies reported a measure of SB as an outcome variable. A total of 38% (3/5) of the studies reported a reduction in SB, whereas 12% (1/8) of the studies [40] reported no change in sitting time and 12% (1/8) [38] reported an increase in SB. Although Blake et al [38] reported an increase in SB, they did report a significantly lower increase in SB in the intervention group than in the control group.

When reporting the physical behavior outcomes, most studies (5/8, 62%) reported PA or SB during work hours, during which the intervention took place [18,35-37,40], whereas 25% (2/8) of the studies reported PA [16,38] and SB [38] as minutes or hours per week as the intervention could be continued outside of work hours. Furthermore, 12% (1/8) of the studies provided measures of PA daily (steps) and over a week (walking days per week) [39]. Finally, Parry et al [18] reported the physical behaviors as a percentage of accelerometer wear time for work hours and over the workday, and Mackenzie et al [35] provided the SB data during work hours in total and split into morning and afternoon.

The intervention outcomes reported in the included studies related to PA and SB are further outlined in Table S3 in Multimedia Appendix 1.

#### Reported Benefits of Taking a PR Approach

Many of the included studies (5/8, 62%) provided some context and evaluated how taking a PR approach benefited or hindered the research process and alluded to barriers that were experienced by the participants or researchers.

In total, 25% (2/8) of the studies stated that the PR approach used was the key component to the success of the WHP intervention [18,38], whereas 38% (3/8) of the studies stated that taking a PR approach benefited the development of the intervention [18,35,36]. The authors noted that taking a PR approach and including the participants’ views and opinions ensured that the interventions were suitable to the workplace surrounding and, therefore, more likely to be acceptable and feasible for employees [18,35,36]. Mackenzie et al [35] hailed the PR approach for allowing for the development of a “real-world” pragmatic intervention. This point was expanded on further by Griffiths et al [36], who stated that the development group allowed for discussions and refinement of the initial intervention concept, which resulted in an agreed upon evidence-based intervention that was deemed feasible and acceptable by the stakeholders.

Some further benefits of the participatory approach were noted. These were awareness of the intervention [35] and increased communication about PA, suggesting that the information reached the workforce [40]. Managers’ behavior was noted as a motivating factor for employees to sit less and move more [40], and peer support was also identified as an important facilitator of intervention adoption [38]. Participants expressed that they had positive experiences about senior management as they offered great organizational support for the duration of the project and designed and developed the study’s promotional posters, which were regarded as high quality [38].

#### Reported Barriers of Taking a PR Approach

Despite the several benefits highlighted by the included studies, some barriers were also noted, such as lack of peer support, which reduced engagement [38]; lack of support from management and team leaders [18,38]; and lack of awareness of the intervention components (ie, workplace champions and Twitter updates) rendering them unhelpful to the participants [35]. Furthermore, Parry et al [18] reported limited success in changing the organizational culture in workplaces even when management and participants were aware of the intervention options, leading to suggestions of stronger external support, such as guidelines.

Griffiths et al [36] presented and reported barriers when taking a PR approach. These were (1) inconsistent stakeholder attendance, which reduced stakeholder input in discussions at meetings; (2) conducting focus group meetings on the web rather than in person as this was thought to lose some of the possible interaction among stakeholders; (3) the fact that attempting to align the research with reality required compromise from both the stakeholders and research team, which did not guarantee resolution for all stakeholders; and (4) the fact that having a small sample from a few different organizations in the development group and piloting of the intervention facilitated more in-depth discussion and greater input from participants but that whether the intervention translated to other organizations and, subsequently, its applicability and adherence were unknown [36].

A total of 38% (3/8) of the included studies did not evaluate how the PR approach may have benefited or hindered the WHP intervention or its outcomes [16,37,39].

## Discussion

### Summary of Evidence: End User Engagement

This scoping review addressed the research question and objectives, which were to identify and understand how previous WHP studies implemented PR with desk-based office workers. The systematic search retrieved 8 studies that met our inclusion criteria. Overall, there is evidence suggesting that taking a PR approach is beneficial to the development of a WHP intervention [18,35,36] and a key component of its success [18,38]. The main reasons cited in the included studies were that, by taking a PR approach, the participants’ opinions and lived experiences were included, which ensured that the intervention was suitable for the workplace and, ultimately, feasible and acceptable to the end users and relevant stakeholders [18,35,36].

A defining characteristic of PR is the degree of engagement and power shared with the end users during the different stages of the research process and decision-making more so than the methods and techniques used [8,56]. A key finding from this scoping review was that, among the 8 identified WHP studies that incorporated end users, the inclusion of the participants ended at the conception of the WHP intervention, with 2 (25%) studies not involving the end users or stakeholders in their conception and only in assisting with the implementation and facilitation of the study [38,40]. An important point to highlight is that, while we use the term *conception of the WHP intervention*, there was a distinct lack of engagement of end users in the conception of the overall study (ie, none of the included studies commenced due to an end user highlighting their occupational physical behaviors as a health priority and seeking an academic partner). In fact, all the included studies commenced with an academic institute or partner approaching an organization or organizations and recruiting participants and end users to the study before then developing the WHP intervention in collaboration with the end users and relevant stakeholders at varying levels of involvement and shared decision-making. In PR, the research problem should ideally originate from the community or end users [24]; however, in reality, we find that most PR projects are in fact initiated by researchers. Therefore, participatory processes are actively applied to shift ownership and control of the research process toward the end user community [57]. This leads to a more equitable and democratized decision-making process to facilitate the shift in knowledge leadership and support community ownership of the research process and developed intervention [57].

The degree of engagement and power that was shared with end users was evaluated to be low, with 25% (2/8) of the studies determined to be nonparticipation studies [38,40] and 25% (2/8) determined to be tokenistic in how the end users were involved in conceiving and designing the WHP studies [15-34,41-49]. This finding is similar to that of another identified review, which investigated studies that took a participatory action research approach to promote mental health and resilience in youth and adolescents [58]. Raanaas et al [58] identified a distinct lack of authentic involvement of the end users in their included studies, which is similar to our own included studies, with only 38% (3/8) of the studies determined to have provided citizen power at the lowest rung (“partnership”) [18,35,36]. The similarities, despite the differences in population and outcome measures, can largely be attributed to the PR approach taken by the researchers and the power they shared with the end users.

The evaluation of the included studies via the ladder by Arnstien [27] placed an important emphasis on the level of involvement and shared decision-making, so the evaluation and final “labeling” of the included studies was not impacted by the lack of involvement in the later stages of the research process (data collection, analysis and reporting, and dissemination). However, the evaluation and, therefore, “labeling” was influenced by how the participants were involved in the development of the subsequent WHP intervention, with the included studies labeled as nonparticipatory stating that the research team developed the study without involvement of the end users or relevant stakeholders [38,40] and 25% (2/8) of the studies being labeled as tokenistic as evidenced by the research team tailoring the intervention components to the end users but retaining the overall decision-making power by (1) reaching a consensus from ideas generated by the workers [37] and (2) having a fixed prescribed exercise break of 5 to 10 minutes, which was instructed by the research team [16]. Therefore, 50% (4/8) of the studies demonstrated a lower level of involvement and shared decision-making, as opposed to the 50% (4/8) of the studies that were determined to provide citizen power, which noted a compromise or generated all ideas with the end users. Instead of excluding, tailoring, or retaining the right to draw the conclusions from a consensus of end users’ lived experience.

A goal in PR is that end users should “own” the research process [8], but in this review, we identified a glaring lack of end user involvement in the overall research process. When using the phrase “own the research," the authors of this scoping review refer to the shift in knowledge leadership and influence from the researcher or academic institute to the community or workplace, which then has ownership and self-determination over the research process and subsequent intervention or interventions [57]. None of the included studies included engagement strategies such as creating a stakeholder board (beyond an advisory board) or council or involving end users as coresearchers in data analysis or research dissemination. These findings are bleak and identify an issue in this emerging field of PR in office-based WHP. One potential reason for the lack of authentic involvement observed in this review may be that 38% (3/8) of the studies were pilots [35-37]. However, authentic involvement was also absent in the remaining 62% (5/8) of the studies, and therefore, future research should strive to move beyond using PR to tailor and implement an intervention toward involving end users in the entire research process.

### Summary of Evidence: Methods of Involving the End Users and Relevant Stakeholders

In this scoping review, we aimed to discuss how end users and relevant stakeholders were involved and how the participants’ involvement shaped the design of the WHP intervention. To address this aim, we conducted a thematic analysis and charted extracted data on the methods of PR (Table S2 in Multimedia Appendix 1). When conducting the thematic analysis to examine the role of the end users and the level of involvement in the coproduction of the WHP studies, we identified 4 primary themes. The four themes were (1) participant workshops, focus groups, meetings, or brainstorming sessions; (2) manager or management involvement; (3) researcher facilitator; and (4) workplace champions, health inspirers, team leaders, or health strategists. These themes can be compared to the set of themes identified in a previous broader review of end user involvement strategies [59].

The key findings from the thematic analysis in relation to the methods of involving end users were that the included studies invited end users to attend an initial focus group meeting where they were asked to provide their opinions and personal experiences in relation to designing or evaluating a WHP intervention. These focus group meetings were often cited as the conception of strategies that formed or were implemented alongside the WHP intervention. However, this was also often the end of the end user involvement, meaning that the focus groups may have been conducted in a tokenistic manner or tick-box exercise for an intervention that had already been largely planned. Focus groups in and of themselves do not constitute participatory engagement but are simply another form of data collection, which Arnstein [27] rates as tokenistic consultation. One of the included studies noted that compromises were made by the research team, suggesting that power was shared in the decision-making and development of the WHP intervention [36].

Another key finding from the thematic analysis and evaluation of the end users’ level of participation was the use of elevated positions that were created by the research team in the included studies, such as “team leaders,” “health inspirers,” and “workplace champions.” These elevated positions were created and asked end users to take on these positions and lead by example, support, and facilitate the delivery of different intervention components and in some cases provide more in-depth insights or participate in the codevelopment of intervention components [40]. However, barring the latter point, the use of elevated positions is not uncommon in nonparticipatory interventions, which may use members of a research team, intervention facilitators, or participants to assist in the facilitation of an intervention. Whereas a benefit of taking a PR approach is that end users in these elevated positions will remain engaged upon completion of the study and researcher involvement. Thus, these end users in elevated positions have an increased capacity to continue supporting behavior change within the community or workplace.

It is important to note that the lack of reporting on the PR approaches taken in the included studies restricts the conclusions that can be drawn as to whether the end users involved in the conception of the intervention were involved as the intervention facilitators. In some of the included studies (2/8, 25%), it was evident that other or additional employees and not solely the end users involved in intervention design were asked to be the intervention facilitators [38,39]. The potential impact of this is unknown regarding whether it would be a benefit or detriment to the facilitation of the intervention components. The reasons for this were not reported in the included studies but may be related to end user burden, to the end user involved in the PR approach not being an eligible participant, or to increased intervention facilitators being needed when the intervention is rolled out across a large workplace compared to the number of individuals (end users and stakeholders) involved during the commencement of the PR approach and conceptualization.

### Summary of Evidence: Effect of PR WHP Interventions on PA and SB

An objective of this scoping review was to discuss the evaluation and outcomes of the included studies. Overall, the PR approach was shown to increase PA in 86% (6/7) and decrease SB in 60% (3/5) of the studies that measured these outcome variables, respectively. These changes were typically reported during occupational hours, which is when the interventions were generally prescribed. Only 12% (1/8) of the studies noted an increase in SB; however, the increase in the intervention group was significantly lower than the increase in the control group, suggesting that the PR approach may have blunted the increase in SB, whereas the final study that investigated SB noted no change. Caution should be taken when interpreting these findings as 38% (3/8) of the included studies [35-37] were pilot studies with no control comparator groups and small sample sizes (ie, not powered to detect a change in physical behavior [PA or SB]). However, the findings of this scoping review and of the included studies do suggest that PR should be investigated further as a research approach within health research due to the numerous benefits identified and the positive results observed overall.

### Implications

The included studies and this scoping review provide insights into how PR has been incorporated in office-based PA WHP studies by reporting and evaluating the methods, the degree of engagement and power shared with end users, and the barriers to and benefits of taking a PR approach in the workplace setting. This review identified a lack of authentic and meaningful involvement of the end user, where they were asked to provide input to tailor an intervention but were not provided with the opportunity to “own” the research process. Future research should move beyond piloting PR in WHP interventions and start from the workplace to actively collaborate, identifying the end users’ priorities and developing or tailoring an intervention collaboratively with them to address their priorities. Further recommendations would be to (1) include end users in the published articles, presentations, and reports; and (2) build capacity within the workplace with a stakeholder board, management, or workplace champions that empower the end users and create a sustainable WHP intervention after researcher involvement. (3) A key recommendation for future WHP studies or any study that wishes to take a PR approach would be to allow adequate time for the coconstruction or collaboration to occur and opportunity for decision-making to be shared and, therefore, for trust to be built [60] between the community or workplace and the research team. Some of the included studies (2/8, 25%) conducted a single “participatory” event, workshop, or focus group, which was often done in a tokenistic manner and to tailor a planned intervention from academia and not from the community. Ultimately, the act of conducting a single “participatory meeting” means that the researchers retain the power or ownership of the research process and decision-making of the research project and do not provide an equitable environment. Researchers should step outside the “normal” confines of research and share power and resources and develop capacity within their relevant community and end users to allow them to appropriately engage in all stages of the research process. This could be in the form of a larger number of participatory workshops (≥3 meetings), earlier engagement with end users to help conceptualize a suitable intervention that addresses their identified health priorities, and training and involvement in all aspects of the research process to increase the capacity of the included end users. These recommendations may facilitate the shift in knowledge leadership and encourage the sharing of power and movement toward end user ownership and self-determination over the research process and subsequent intervention or interventions.

### Limitations

While this is the first scoping review to examine how PR is being incorporated in PA WHP studies in desk-based adults working in offices, in which we identified a multitude of common traits regarding how participants and the end users were involved, some limitations should be considered.

When evaluating the degree of participation and power shared between participants and researchers in the WHP studies, we used the ladder of citizen participation by Arnstein [27]. Although the ladder by Arnstein [27] is well regarded, our interpretation of the included studies relied solely on self-reported end user involvement details in published literature and associated documents. Reporting standards for patient and public involvement are generally not well followed [61]; therefore, we could only evaluate the studies based on limited reporting, which may have missed details of engagement and shared power. This echoes the limitation of Frankena et al [62], who conducted a structured literature review and found it difficult to evaluate from written text whether the inclusion of their relevant end users (people with intellectual disabilities) was meaningful or tokenistic. Frankena et al [62] stated that more information was often needed regarding the process of inclusion.

### Conclusions

In conclusion, the findings from this scoping review provide a foundation of evidence for how PR is currently being implemented in office-based PA WHP studies. We observed that the end user is currently only incorporated in the conception and implementation of the WHP studies and that, largely, the studies were tokenistic or nonparticipative, whereas 50% (4/8) of the studies were evaluated to provide citizen power in the conception of the interventions. Overall, a benefit was observed with positive improvements in PA and reductions in SB across the studies, which was largely attributed to taking a PR approach and involving the end users, which allowed for the design of a WHP intervention that was feasible and acceptable. Future studies should aim to move beyond a pilot and feasibility trial and collaborate with the workplaces, building capacity and empowering the workforce by providing citizen control and letting the end users “own” the research for a sustainable WHP intervention after researcher involvement.

## Appendix

Multimedia Appendix 1 Participatory research methods, intervention components, and reported outcomes of the interventions in the included studies in relation to physical activity and sedentary behavior and completed PRISMA-ScR (Preferred Reporting Items for Systematic Reviews and Meta-Analyses extension for Scoping Reviews) checklist.

## Abbreviations

PA: physical activity
PICO: population, intervention, comparison, and outcome
PR: participatory research
PRISMA: Preferred Reporting Items for Systematic Reviews and Meta-Analyses
PRISMA-ScR: Preferred Reporting Items for Systematic Reviews and Meta-Analyses extension for Scoping Reviews
SB: sedentary behavior
WHP: workplace health promotion

## Data Availability

Data sharing is not applicable to this paper as no data sets were generated or analyzed during this study.
