# Supplementary material for: Participatory Approaches in the Context of Research Into Workplace Health Promotion to Improve Physical Activity Levels and Reduce Sedentary Behavior Among Office-Based Workers: Scoping Review
Source: JMIR Public Health Surveill. 2024 Jun 19;10:e50195. doi: 10.2196/50195 (PMC11222769; doi:10.2196/50195)
Supplement: Multimedia Appendix 1 [file publichealth_v10i1e50195_app1.docx]

**Multimedia Appendix 1**

**Title: Participatory Approaches in the Context of Research into Workplace Health Promotion to Improve Physical Activity Levels and Reduce Sedentary Behaviour among Office-Based Workers: Scoping Review**

**Authors:** Aidan John Buffey^1,2^, Christina Kate Langley^3^, Brian P. Carson^1,2^, Alan E. Donnelly^1,2^, and Jon Salsberg^4,5^

**Affiliations:**

^1^Department of Physical Education and Sport Sciences, Faculty of Education and Health Sciences, University of Limerick, Limerick, Ireland

^2^Physical Activity *for* Health Research Cluster, Health Research Institute (HRI), University of Limerick, Limerick, Ireland

^3^University Academy 92, Old Trafford Campus, Manchester, United Kingdom

^4^Public and Patient Involvement Research Unit, School of Medicine, University of Limerick, Limerick, Ireland

^5^Public and Patient Involvement Research Unit, Health Research Institute (HRI), University of Limerick, Limerick, Ireland

**Corresponding Author:** Aidan John Buffey (Aidan.Buffey@ul.ie)

**Multimedia Appendix 1: Table S1. PRISMA-ScR (Preferred Reporting Items for Systematic reviews and Meta-Analyses extension for Scoping Reviews) Checklist**

| **SECTION** | **ITEM** | **PRISMA-ScR CHECKLIST ITEM** | **REPORTED ON PAGE #** |
| --- | --- | --- | --- |
| **TITLE** | | | |
| Title | 1 | Identify the report as a scoping review. | 1 |
| **ABSTRACT** | | | |
| Structured summary | 2 | Provide a structured summary that includes (as applicable): background, objectives, eligibility criteria, sources of evidence, charting methods, results, and conclusions that relate to the review questions and objectives. | 1-2 |
| **INTRODUCTION** | | | |
| Rationale | 3 | Describe the rationale for the review in the context of what is already known. Explain why the review questions/objectives lend themselves to a scoping review approach. | 3 |
| Objectives | 4 | Provide an explicit statement of the questions and objectives being addressed with reference to their key elements (e.g., population or participants, concepts, and context) or other relevant key elements used to conceptualize the review questions and/or objectives. | 3 |
| **METHODS** | | | |
| Protocol and registration | 5 | Indicate whether a review protocol exists; state if and where it can be accessed (e.g., a Web address); and if available, provide registration information, including the registration number. | 3: Methods; “Protocol and Registration” |
| Eligibility criteria | 6 | Specify characteristics of the sources of evidence used as eligibility criteria (e.g., years considered, language, and publication status), and provide a rationale. | 3  4: Textbox 1 |
| Information sources* | 7 | Describe all information sources in the search (e.g., databases with dates of coverage and contact with authors to identify additional sources), as well as the date the most recent search was executed. | 4 |
| Search | 8 | Present the full electronic search strategy for at least 1 database, including any limits used, such that it could be repeated. | 4  (Reference to the protocol manuscript where the full electronic search strategy for each of the five searched electronic databases can be found, including any limits) |
| Selection of sources of evidence† | 9 | State the process for selecting sources of evidence (i.e., screening and eligibility) included in the scoping review. | 4-6 |
| Data charting process‡ | 10 | Describe the methods of charting data from the included sources of evidence (e.g., calibrated forms or forms that have been tested by the team before their use, and whether data charting was done independently or in duplicate) and any processes for obtaining and confirming data from investigators. | 6 |
| Data items | 11 | List and define all variables for which data were sought and any assumptions and simplifications made. | 6  (Reference to protocol manuscript where a detailed table and associated question for each data item is presented) |
| Critical appraisal of individual sources of evidence§ | 12 | If done, provide a rationale for conducting a critical appraisal of included sources of evidence; describe the methods used and how this information was used in any data synthesis (if appropriate). | N/A  6 (Stated that a critical appraisal was not conducted) |
| Synthesis of results | 13 | Describe the methods of handling and summarizing the data that were charted. | 10-11 |
| **RESULTS** | | | |
| Selection of sources of evidence | 14 | Give numbers of sources of evidence screened, assessed for eligibility, and included in the review, with reasons for exclusions at each stage, ideally using a flow diagram. | 5: Figure 1 (PRISMA flow diagram)  7 (Narrative synthesis) |
| Characteristics of sources of evidence | 15 | For each source of evidence, present characteristics for which data were charted and provide the citations. | 7-9  Main Manuscript: Table 1 (Page 8-9)  Multimedia Appendix 1: Table S2 and Table S3 |
| Critical appraisal within sources of evidence | 16 | If done, present data on critical appraisal of included sources of evidence (see item 12). | N/A |
| Results of individual sources of evidence | 17 | For each included source of evidence, present the relevant data that were charted that relate to the review questions and objectives. | 8-9: Table 1  11-12: Table 2  Multimedia Appendix 1: Table S2 and Table S3 |
| Synthesis of results | 18 | Summarize and/or present the charting results as they relate to the review questions and objectives. | 10-15  Multimedia Appendix 1: Table S2 and Table S3 |
| **DISCUSSION** | | | |
| Summary of evidence | 19 | Summarize the main results (including an overview of concepts, themes, and types of evidence available), link to the review questions and objectives, and consider the relevance to key groups. | 15-17 |
| Limitations | 20 | Discuss the limitations of the scoping review process. | 17 |
| Conclusions | 21 | Provide a general interpretation of the results with respect to the review questions and objectives, as well as potential implications and/or next steps. | 17 |
| **FUNDING** | | | |
| Funding | 22 | Describe sources of funding for the included sources of evidence, as well as sources of funding for the scoping review. Describe the role of the funders of the scoping review. | 17 |

**Multimedia Appendix 1: Table S2. Displays each of the included studies methods of participatory research and how they engaged with end-users and relevant stakeholders. This table illustrates the method, number of sessions of the method(s) and duration, number of participants engaged in each method and the content/agenda discussed when meeting end-users and relevant stakeholders.**

| **Study and (Year)** | **Participatory Research Methods** | **Number of Sessions and Duration** | **Number of Participants** | **Content/Agenda** |
| --- | --- | --- | --- | --- |
| Blake et al (2019) [38] | Organisational committee,  responsible for:   - Internal marketing   - Branding the intervention   - Co-developed logo, promotional posters and intervention webpage - Delivery of orientation - Motivation briefing sessions for the team leaders   Group-based engagement with the intervention and co-workers involved in provision of support for PA.  Team leaders acted as intervention facilitators; the role of the team leaders was to:   - Facilitate intervention delivery - Encourage co-workers to engage with the intervention - Be a contact for participants to discuss challenges and barriers with the Qigong challenges - Model the sequence of movements during each set exercise break session - Co-creation of the video materials modelling the exercises (six videos of two minutes each)   Employee volunteers who assisted with:   - Technical support - Production of materials - Exercise demonstrations   Stakeholder consultation | The general manager and HR manager delivered a standard 30-minute orientation and motivation briefing session to all team leaders before the intervention began. | Organisational committee; n=4:   - Two team leaders - Two HR officers   Team leaders (n=31)  Stakeholder consultation: members of the participating organisations, the Qigong Master and the project team. | The organisational committee was set up to develop and implement company policy on the internal marketing of the intervention.  Stakeholder consultation: To establish the duration and content of the training session for team leaders/intervention delivery. |
| Gilson et al (2016) [37] | Participatory workshop | One workshop for one-hour, held at the worksite (June 2014). | n=10-15 | The workshop followed the baseline measures.  In the workshop, the researchers reviewed evidence on the benefits of reducing sitting and increasing physical activity. The workers were asked to identify and discuss occupational strategies for ‘sitting less and moving more’.  The aim of the participatory workshop was to identify occupational strategies for “sitting less and moving more”. |
| Griffiths et al (2022) [36] | Co-production development focus groups (between June 2020 and March 2021).   - Conducted in small groups (n=4-6) - Facilitated by the researcher using open questions and asking for alternative viewpoints. | A prior/initial needs analysis (online questionnaire) was conducted before participants opted in participate in the development group.  Online focus group 1 (95-minutes):  Online focus group 2 (65-minutes):  Intervention piloting:  Online focus group 3 (45-minutes): | n=11  n=11   - Conducted in groups of 4-6 but not certain/clear on the number/breakdown between focus groups.   Conducted with the pilot group (n=5)  Conducted with the pilot group (n=5)  Conducted with the pilot group (n=5) | Data from the needs analysis was thematically analysed and summarised by the research team to be used as focal discussion points within the focus groups.  Presentation and education on the importance of breaking up sitting time regularly for cardiovascular health before opening the discussion to the concept of breaking up sitting time and initial perceived challenges. Finally, they were also asked to comment and discuss how they would prefer to break up sitting time. A note area was publicly visible throughout.  Participants built upon the previous meeting by discussing and commenting on details of the intervention, ie. the intervention components (how long, how often and the modality) and intervention support (eg. daily reminders). The intervention and support were summarised in the conclusion of the focus group.  Participants who completed the intervention pilot met to discuss the challenges and facilitators of the intervention and intervention support, with the use of an online flip chart. Proposed solutions were openly discussed before concluding the participatory process and proposed intervention refinements.  For further information on the content of this included study please refer to Griffiths et al [42] who provide a summary of the co-production participatory process and its associated objectives in the supplementary table (Table S1). |
| Kong et al (2022) [39] | An employee advisory board. | No information provided. | n=4 to 7 employees from all occupational sectors in the worksite. | The employee advisory board was established to work closely with the research team to design and implement intervention activities. |
| Mackenzie et al (2015) [35] | An intervention development focus group and email suggestions from those unable to attend. | One focus group for one-hour. | n=7/11 attended the focus group.  n=4/11 submitted suggestions via email. | The framework for the focus group was taken from previous published literature by Dunstan et al. (2013):  This involved an initial description of the associations between prolonged sitting and health.  Followed by a “brainstorming” session where strategies were identified by participants on how to reduce workplace sitting time. |
| Parry et al (2013) [18] | Each workplace (three) was asked to attend two structured meetings at their workplace to discuss and develop their specific workplace intervention.  Team leaders (little to no-information provided)  Just stated that the facilitator (lead researcher (S.P.) communicated with team leaders and management to help with implementation. | Two structured meetings (no duration provided):  The first structure meeting (no duration provided).  ‘Homework’ between meetings.  The second structured meeting (2-3 weeks) following the first meeting (no duration provided). | The structured meetings were run by the Lead Research (S.P.) as a facilitator.  All participants were invited, no figure was provided for attendance to the structured meetings opposed to number of participants. | Meeting 1:  “Brains-stormed” ideas/options to promote their specific intervention (active office, PA or office ergonomics).  ‘Homework’ (between meetings):  Participants were encouraged to think and refine specific workplace intervention strategies.  Meeting 2:  Participants were asked to share their ideas and rated the potential strategies in terms of feasibility and effectiveness.  In Meeting 2, an action plan was developed and the lead researcher/facilitator (S.P.) communicated with team leaders and management to help implementation. |
| Tan et al (2016) [16] | Participatory (as named by the study) workshops. | Three participatory workshops over six weeks (no duration provided). | All participants assigned to the intervention group. | Each participatory workshop focused on participatory skill-building activities, peer support. Goal-setting exercises and problem-solving discussions to attain individual goals and overcome individual barriers.  These workshops had a strong focus on behavioural strategies guided by Bandura’s Self-Efficacy.  The workshops for diet and PA were unique in the nature and design of their activities, due to the different entities requiring different behavioural strategies. |
| Wahlstrom et al (2019) [40] | Participatory approach with collaboration between researchers and workplace representatives.  Workshops | No detail provided on the number or duration of these collaborative meetings.  Four workshops (no duration provided) | When planning the relocation of participants to a new office, organisational representatives, the researchers and an ergonomist from the occupational health service.  ‘Health inspirers’ (n=2-7) and a health strategist. | Discussed how the interior design could be further developed to facilitate PA in the office.  The communication campaigns were developed which involved brainstorming and discussion among attendees. |

Abbreviations: PA: physical activity; HR: human resources; mins: minutes.

**Multimedia Appendix 1: Table S3. Presenting the intervention components and reported outcomes of the intervention in relation to physical activity and sedentary behaviour of each included study.**

| **Study** | **Year** | **Intervention Components** | **Intervention Outcomes** |
| --- | --- | --- | --- |
| Blake et al [38] | 2019 | 1. Digital intervention featuring a series of six video clips demonstrating “Qigong” exercises, undertaken twice per day for 10-minutes on every working day at set exercise break times. 2. “Qigong” is an ancient Chinese exercise that involves deep abdominal breathing and stretching. 3. Each participants computer had a “Move-It” icon that was scheduled to pop up twice a day, at the same time every day: 10:50 and 15:50. This acted as a sign-in and a prompt for the participants to interrupt prolonged sitting and to individually perform the exercise routines (individual level activity) beside their usual workstation. 4. Participants could choose to participate in an exercise group led by their team leader at the allocated times, or to ignore the prompt and participate individually at their workstations guided by a video at a time of their preference. 5. Promotional posters and exercise videos were placed in high visibility areas including office corridors and the staff canteen with the promotional videos being shown on a large screen in the canteen. 6. Team leaders. | **Physical Activity (Expressed as Hours/Week):**   1. PA (expressed as hours/week) increased in the control group from T1 (18.75 ± 19.75) to T2 (27.20 ± 30.84) (Changes Intercepts: 7.41 ± 3.64, *p* = .04). 2. PA (expressed as hours/week) increased in the intervention group from T1 (22.87 ± 21.84) to T2 (27.75 ± 25.98) (Changes Intercepts: 5.80 ± 2.00, *P* < .001). 3. The control group achieved a greater increase in PA (expressed as hours/week) compared to the change achieved by the intervention group (T1 vs. T2). The difference (-1.61 ± 4.16 (95% CI: -9.76, 6.53) did not reach statistical significance (*P* = .70, *d* = 0.05).   **Sitting Hours (Expressed as Hours/Week):**   1. Sitting hours (expressed as hours/week) increased in the control group from T1 (9.20 ± 2.20) to T2 (9.41 ± 2.0) (Changes Intercepts: 10.34 ± 1.04, *P* < .001). 2. Sitting hours (expressed as hours/week) increased in the intervention group from T1 (9.51 ± 2.69) to T2 (9.64 ± 2.55) (Changes Intercepts: 5.68 ± 0.82, *P* < .001). 3. Despite both the control and intervention group significantly increasing their sitting hours post intervention compared to pre-intervention. The mean difference was significantly lower in the intervention group compared to the control group (-4.66 ± 1.32 (95% CI: -7.25, -2.08, *P* < .01, *d* = 0.44).   **Work Performance:**   1. The control group self-reported a significant increase in work performance post-intervention (T2; 7.50 ± 1.75) compared to pre-intervention (T1; 7.63 ± 1.40). The Changes Intercepts were 7.41 ± 3.64, *P* = .01. 2. There were no significant changes for self-reported work performance in the intervention group when comparing T1 (6.89 ± 1.41) vs. T2 (6.96 ± 1.52). The Changes Intercepts were -0.03 ± 0.12, *P* = .78. 3. The control group achieved a significant increase in self-reported work performance post intervention compared to pre-intervention whereas this was not observed in the intervention group. The mean difference between the control and intervention group was -0.72 ± 0.27 (95% CI: -1.25, -0.19, *P* = .01, *d* = 0.36) showing the control group achieved a greater increase in work performance. |
| Gilson et al [37] | 2016 | 1. **Intervention Protocol 1 (IP1):** Implemented ‘sit less and move more’ strategies without prompts from the software package and [Sitting Pad]. 2. **Intervention Protocol 2 (IP2):** Implemented ‘sit less and move more’ strategies and software [Sitting Pad] fitted to the participants office chair that provided real time, reactive feedback, and prompts. The prompts moved from green to amber to red after 30 and then 60-minutes of continuous desk sitting. The software would reset to green after five continuous minutes of the Sitting Pad not being activated. | GENEActiv data presented as a percentage of worktime as mean ± SD and Sitting Pad data presented as min/day, presenting baseline, end-intervention, and changes (end intervention – baseline) for work time SB, PA and desk sitting relative to intervention protocol.   1. **IP1:** Relative to baseline, end-intervention GeneActiv data showed that participants reduced their sedentary work time by an average of 2% (18 mins/day) (Baseline: 68 ± 14, End Intervention: 66 ± 15, *P* > .05). 2. **IP2**: Relative to baseline, end-intervention GeneActiv data showed that participants reduced their sedentary work time by an average of 8% (72 mins/day) (Baseline: 74 ± 7, End Intervention: 66 ± 15, *P* = .012). 3. **IP1:** Light (%) GENEActiv increased by 1% from baseline (Baseline: 25 ± 11, End Intervention: 26 ± 12, *P* > .05). 4. **IP2:** Light (%) GENEActiv increased by 8% from baseline (Baseline: 19 ± 7, End Intervention: 27 ± 14, *P* < .01). 5. **IP1:** Moderate+ (%) GENEActiv increased by 1% from baseline (Baseline: 7 ± 4, End Intervention: 8 ± 5, *P* > .05). 6. **IP2:** Moderate+ (%) GENEActiv remained stable from baseline (Baseline: 7 ± 3, End Intervention: 7 ± 3, *P* > .05). 7. **IP1:** Total time sitting increased by 10 minutes from baseline (Baseline: 370 ± 84, End Intervention: 380 ± 81, *P* > .05). 8. **IP2:** Total time sitting decreased by 13 minutes from baseline (Baseline: 372 ± 53, End Intervention: 359 ± 71, *P* > .05). 9. **IP1:** Longest bout sitting increased by 17 minutes from baseline (Baseline: 100 ± 42, End Intervention: 117 ± 49, *P* > .05). 10. **IP2:** Longest bout sitting decreased by 15 minutes from baseline (Baseline: 111 ± 45, End Intervention: 96 ± 30, *P* > .05). 11. **IP1 vs. IP2:** When comparing the mean longest sitting bout recorded in IP1 against IP2, there was a significant 32 min/day difference (*P* = .018). |
| Griffiths et al [36] | 2022 | 1. Breaking up sitting time hourly with five-minutes of light-paced walking by the desk during working hours. 2. Daily email reminders (prompts) highlighting the importance of breaking up sitting time. | Data gathered between work hours was grouped and averaged and presented graphically with some means discussed descriptively, we present the reported descriptive results here as means.   1. The number of sedentary breaks significantly increased from 2 pre-intervention, to 10 (Week 1, *P* = 0.01) and 11 (Week 2, *P* = .04). 2. There were no changes in daily average calories expended between weeks (*P* = .642). 3. There were no changes in average daily MVPA between weeks (Week 1: 100 min/day, Week 2: 109 min/day, Week 3: 89 min/day, *P* = .583). |
| Kong et al [39] | 2022 | Individual Level:  The goals at the individual level were to improve the employees’ health awareness, compliance, and skills to increase their physical activities and healthy food consumption.   1. Six lectures, with one lecture provided every two months from January 208 to November 2018 covering: weight management, healthy diets, physical activities and health preservation of traditional Chinese Medicine. 2. Walking routes and activities suitable for the workplaces were designed and open to all employees in each worksite to improve access to physical activities for employees. 3. Health information about weight-related lifestyle changes was pushed by the WeChat subscription (“Official Health Management”) two-three times weekly. Furthermore, the dietary energy and energy consumption of various sports provided throughout the intervention period was provided. 4. Daily steps were automatically ranked and distributed by the WeChat Subscription of the study and material (extrinsic) rewards were provided by the worksites to enhance employees’ enthusiasm to participate in the activities. 5. Several diet and exercise teams were set up on a voluntary basis, based on employees’ interests and personal relationships to monitor and improve their daily behaviours by recording the daily diet or exercise of team members and remind each other. Team members were asked to record their daily diet or exercise.    1. Members of the diet teams supervised what each other ate and drank during meals and snacks, and any person with unhealthy food would be reminded by others on the spot.    2. Members of the exercise teams would exercise regularly at an agreed time and place. For example, the members of the walking team reminded each other after lunch every day and walked together along a set route.   Environmental Level:  The goals at the environmental level were to create a positive environment at workplaces and increase the employees’ access to a healthy diet and physical activities.   1. A kick-off event was held at each worksite to introduce the program to the employees. 2. Posters about physical activities, diets and health were displayed on the bulletin board at the worksites. 3. Signs encouraging walking were posted beside elevators and stairs at workplaces. 4. The “Award for healthy diet showing” and “Exercise challenge” activities were launched for all employees at worksites based on WeChat Subscription to facilitate/promote healthy diet and physical activities maintenance. 5. Food models and scales were placed at the gate, restroom and conference room. 6. Improvement of the dining area and snack bar environment of the worksites. The food calories were displayed in the dining area. The supplies of healthy food were increased including fruit, milk and waters while the supplies of high-calorie food such as instant noodles, French fires and Coca Cola were reduced in the snack bar of worksites. A discount was also encouraged to offer to staff on healthy food. 7. A fitness area was set up at each worksite where some sport equipment was provided freely for the employees.   Organisational Level:   1. An employee advisory board (EAB), which consisted of four to seven employees from all occupational sectors in the worksite, was established in each worksite and worked closely with the research team to design and implement intervention activities. 2. Positive policies were created in worksites including food subsidy policies for snack bars, financial policies for clubs and groups of dietary and activities and reward policies for the “Exercise challenge” and “Show healthy diet and win prizes”. | Physical activity was assessed via self-report measures (Godin-Shephard Leisure Time Physical Activity Questionnaire (GSLT-PAQ; International Physical Activity Questionnaire (IPAQ) and pedometers (Omron HJ-321).  **Godin Index:**  Control:  Baseline: 24.86 ± 29.85  Follow-Up: 23.15 ± 19.48  *P*-Value: 0.88  Intervention:  Baseline: 22.67 ± 20.04  Follow-Up: 25.77 ± 27.98  *P*-Value: .18  Adjusted Change between Groups, Mean/OR (95% CI): 1.04  (−3.59, 5.67)  Adjusted *P*-Value: .66  **Walking Days per Week:**  Control:  Baseline: 5.19 ± 2.30  Follow-Up: 5.21 ± 2.19  *P*-Value: .94  Intervention:  Baseline: 5.16 ± 2.12  Follow-Up: 4.84 ± 2.11  *P*-Value: .09  Adjusted Change between Groups, Mean/OR (95% CI): −0.34  (−0.75, 0.07)  Adjusted *P*-Value: .10  **Daily Steps:**  Control:  Baseline: 6986.14 ± 2526.67  Follow-Up: 4994.83 ± 2226.66  *P*-Value: <.01  Intervention:  Baseline: 6554.41 ± 3113.82  Follow-Up: 6919.76 ± 4412.09  *P*-Value: .36  Adjusted Change between Groups, Mean/OR (95% CI): 863.19  (161.42, 1564.97)  Adjusted *P*-Value: .02  **Exercise to Sweating:**  Control:  Baseline: Yes: 89 (54.3%); No: 75 (45.7%)  Follow-Up: Yes: 85 (71.4%); No: 34 (28.6%)  *P*-Value: .03  Intervention:  Baseline: Yes: 117 (56.0%); No: 92 (44.0%)  Follow-Up: Yes: 98 (63.6%); No: 56 (36.4%)  *P*-Value: .02  Adjusted Change between Groups, Mean/OR (95% CI): 0.68  (0.02, 20.57)  Adjusted *P*-Value: .75  **Exercise Self-Efficacy:**  Control:  Baseline: High: 71 (42%); Low: 298 (58%)  Follow-Up: High: 51 (55.3%); Low: 63 (44.7%)  *P*-Value: .58  Intervention:  Baseline: High: 85 (40.5%); Low: 125 (59.5%)  Follow-Up: High: 53 (34.9%); Low: 99 (65.1%)  *P*-Value: .09  Adjusted Change between Groups, Mean/OR (95% CI): 1.91  (1.02,3.60)  Adjusted *P*-Value: .04 |
| Mackenzie et al [35] | 2015 | 1. Individual: Weekly email from management, with changing content per week (i.e., educational YouTube video, links to the ‘reminder’ software, “Top Tips” and link to the University’s wellness programme health checks). 2. Social: Workplace champions who promoted; stand-up/walking meetings/teaching sessions, “incidental” walking (talking not emailing) and lunchtime walks (linked with the University’s wellness programme), varying week by week. 3. Organisational: Email sent from the Dean to introduce the intervention. Management support standing/walking meetings. Management “lead by example” in taking standing/walking meeting and regular breaks. 4. Environmental: Encourage use of different printer, working/having lunch in a different location, meetings in a different location, using a different toilet. Posters next to the lifts/office clocks. | Within group differences in daily workplace sitting time data are presented as mean ± SD, 95% CI.   1. Mean workplace sitting time was reported at 440 ± 79 min/day pre-intervention and 414 ± 80 min/day post intervention (-26 ± 54, 95% CI: -2 to 53). 2. The mean difference in daily workplace sitting time from pre- to post-intervention was -26 ± 54 min/day, which equated to a 6% reduction. 3. Mean workplace sitting time in the AM: 214 ± 42 mins pre-intervention compared to 205 ± 38 mins post-intervention (-9 ± 25, 95% CI: -4 to 22) 4. Mean workplace sitting time in the PM: 226 ± 54 mins pre-intervention compared to 209 ± 55 mins post-intervention (-17 ± 41, 95% CI: -2 to 53). |
| Parry et al [18] | 2013 | Three differing interventions in three different organisations.  **Intervention A (Active Office Work):**   1. Access to a single ‘Active Workstation’ (treadmill or stationary cycle ergometer) with recommendations to be used for short periods several times a day, starting at 10-minutes and building up to 30-minutes per session. Aimed for all participants to have 30-minutes daily access. 2. Standing or exercises. between calls/document processing. 3. Walk and talk meetings. 4. Active emails (personally delivering information. rather than sending an email). 5. Increase incidental activity in and around the workplace (taking longer routes to the printer, scanner, etc).   **Intervention B: Traditional Physical Activity**   1. Pedometer challenge (increase walking during the workday) 2. Promote active transport (walk instead of taking the bus). 3. Walk and talk meetings. 4. Short frequent walk breaks during breaks, lunchtime, to and from work. 5. Increase use of stairs.   **Intervention C: Office Ergonomics (Control)**   1. “Active” sitting: spending some time perching on the edge of the chair, encouraging movement during sitting. 2. Taking breaks from sitting 3. Standing meetings. 4. Use of “piano stool”: reinforcing active sitting. 5. Use of air cushion.   **Organisations:**  Recruitment meetings were held at suburban branches that employed between 100-150 people. Organisational features and the nature of the office work varied between the organisations.  Organisation 1: Was primarily concerned with data processing of large complex files, employees were able to manage their own time and had flexible working hours and breaks.  Organisation 2: Was a call centre that handled calls ranging from less than a minute to more complex calls lasting many minutes. Every 3-4 days employees completed data processing to provide some job variation. In organisation 2, meetings, work hours and work breaks were set by the national office in another city, meaning there was very little autonomy or flexibility. The productivity, call volume and breaks were also monitored and reported on a weekly basis.  Organisation 3: Was a data processing workplace where workers were required to process a certain number of documents per day and at times were required to make calls or assist in a call centre. Work hours and breaks were scheduled on site and were strictly controlled. Productivity and work compliance were monitored. | **Pre-Intervention vs. Post Intervention (Grouped):**   1. There was a significant reduction in sedentary time on workdays, expressed as % of wear time; pre-intervention vs. post-intervention (72.85 ± 7.06 vs. 71.25 ± 7.27; mean change: -1.60, 95% CI: -0.48, -2.72, *P* = .006). 2. There was a significant reduction in sedentary time during work hours (% of wear time); pre-intervention vs. post-intervention (78.29 ± 8.41 vs. 76.6 ± 8.6; mean change: -1.71, 95% CI: -0.37, -3.06, *P* = .014). 3. No significant changes were observed when looking at the percentage of sustained sedentary time (% of wear time), although these did decrease during workdays (mean change: -2.08 (95% CI: -0.47, 4.62), 24.37 ± 12.73 (pre-intervention) vs. 22.29 ± 13.16 (post-intervention), *P* = .108) and work hours (mean change: -3.24 (95% CI: -0.63, 7.11), 28.98 ± 19.34 (pre-intervention) vs. 25.74 ± 18.66 (post-intervention), *P* = .099). 4. The was a significant increase in break rate (number of breaks/sedentary hour) on workdays (mean change: 0.64 (95% CI: 1.08, 0.20), 7.81 ± 2.45 (pre-intervention) vs. 8.45 ± 2.86 (post-intervention), *P* = .005) and work hours (mean change: 0.72 (95% CI: 1.29, 0.15), 6.95 ± 3.20 (pre-intervention) vs. 7.67 ± 3.41 (post-intervention), *P* = .015). 5. Light activity (% of wear time) was significantly increased during work hours post intervention (20.63 ± 7.86) compared to pre-intervention (19.14 ± 7.75), mean change 1.49 (95% CI: 2.87, 0.10), *P* = .036. However, no significant difference was observed when looking at light activity over the whole day (23.85 ± 6.37 (pre-intervention) vs. 24.81 ± 6.48 (post-intervention), *P* = .098). 6. MVPA increased during working hours post intervention but did not reach statistical significance (2.57 ± 1.83 (pre-intervention) vs. 2.79 ± 1.83 (post intervention), mean change: 0.22 (95% CI: 0.69, -0.24), *P* = .334). However, when comparing the working day, MVPA did significantly increase post intervention (3.29 ± 1.83 (pre-intervention) vs. 3.93 ± 2.34 (post intervention), mean change: 0.64 (95% CI: 1.13, 0.14), *P* = .012).   **Intervention Effects Across the Different Organisations:**   1. There were significant differences between organisations at baseline, during work hours: 2. Sedentary Time: F_2,59_ = 3.80, *P*  =  .028 3. MVPA: F_2,59_  =  5.02, *P*  =  .010 4. Break Rate: F_2,59_ = 3.18, *P*  =  .049   After adjusting for baseline measures and type of intervention, pre- to post-intervention changes in sedentary time, sustained sedentary time, light activity, MVPA and break rate during work hours differed by organisation with Organisation 1 responding most to interventions and Organisation 3 responding the least.    **Intervention Effects Across the Different Organisations/Interventions:**  Multivariable linear regression analysis:  Adjusted pre- to post-intervention change (95% CI).  Intervention estimates adjusted for baseline and organisation.  Organisation estimates adjusted for baseline and intervention.  Group Differences in Change (REF – Group) (*ß* (95% CI))  **Sedentary Time – Work Hours (% wear time):**  **Effect of the Different Interventions:**   1. Active Office – A: -3.09 (95% CI: -5.82, -0.35) 2. Office Ergonomics – B: -1.37 (95% CI: -2.86, -0.13) 3. Physical Activity – C: -0.57 (95% CI: -3.54, 2.40)   Omnibus *P*-value for overall group difference = 0.325  **Effect of the Different Organisations:**   1. Organisation 1: -4.07 (95% CI: -6.70, -1.43) 2. Organisation 2: -1.26 (95% CI: -3.32, -0.79) 3. Organisation 3: 0.14 (95% CI: -1.71, 2.00)   Omnibus *P*-value for overall group difference (Intervention) = .325   1. Active Office - A and Office Ergonomics – C: -1.72 (95% CI: -4.94, 1.50), *P* = .289 2. Active Office – A and Physical Activity - B: -2.52 (95% CI: -6.84, 1.80), *P* = .248   Omnibus *P*-value for overall group difference (Organisation) = .043   1. Organisation 1 and Organisation 2: 2.80 (95% CI: -0.75, 6.36), *P* = .120 2. Organisation 1 and Organisation 3: 4.21 (95% CI: 0.66, 7.76), *P* = .021   **Sustained Sedentary Time (Sedentary Bouts >30 mins) – Work Hours (% wear time):**  **Effect of the Different Interventions:**   1. Active Office – A: -2.87 (95% CI: -9.23, 3.49) 2. Office Ergonomics – C: -5.60 (95% CI: -10.29, -0.91) 3. Physical Activity – B: 1.17 (95% CI: -7.24, 9.58)   **Effect of the Different Organisations:**   1. Organisation 1: -8.64 (95% CI: -14.65, -2.64) 2. Organisation 2: -3.84 (95% CI: -9.03, 1.35) 3. Organisation 3: 3.31 (95% CI: 3.49, 10.11)   Omnibus *P*-value for overall group difference (Intervention) = .485   1. Active Office - A and Office Ergonomics – C: 2.73 (95% CI: -5.22, 0.69), *P* = .495 2. Active Office – A and Physical Activity - B: -4.04 (95% CI: -15.55, 7.48), *P* = .486   Omnibus *P*-value for overall group difference (Organisation) = .046   1. Organisation 1 and Organisation 2: 4.81 (95% CI: -2.81, 12.43), *P* = .212 2. Organisation 1 and Organisation 3: 11.95 (95% CI: 2.55, 21.35), *P* = .014   **Light Activity -Work Hours (% wear time):**  **Effect of the Different Interventions:**   1. Active Office – A: 2.53 (95% CI: -0.42, 5.49) 2. Office Ergonomics – C: 1.38 (95% CI: -0.06, 2.81) 3. Physical Activity – B: 0.29 (95% CI: -2.75, 3.33)   **Effect of the Different Organisations:**   1. Organisation 1: 3.57 (95% CI: 0.84, 6.29) 2. Organisation 2: 1.07 (95% CI: -1.12, 3.27) 3. Organisation 3: -0.14 (95% CI: -1.95, 1.68)   Omnibus *P*-value for overall group difference (Intervention) = .616   1. Active Office - A and Office Ergonomics – C: 1.16 (95% CI: -2.23, 4.54), *P* = .497 2. Active Office – A and Physical Activity - B: 2.24 (95% CI: -2.31, 6.80), *P* = .328   Omnibus *P*-value for overall group difference (Organisation) = .124   1. Organisation 1 and Organisation 2: -2.50 (95% CI: -6.26, 1.26), *P* = .189 2. Organisation 1 and Organisation 3: -3.71 (95% CI: -7.30, -0.11), *P* = .044   **Moderate-Vigorous Activity – Work Hours (% wear time):**  **Effect of the Different Interventions:**   1. Active Office – A: 0.97 (95% CI: 0.06, 1.88) 2. Office Ergonomics – C: -0.17 (95% CI: -0.66, 0.31) 3. Physical Activity – B: 0.04 (95% CI: -0.89, 0.98)   **Effect of the Different Organisations:**   1. Organisation 1: 0.69 (95% CI: -0.14, 1.51) 2. Organisation 2: 0.42 (95% CI: -0.28, 1.11) 3. Organisation 3: -0.53 (95% CI: -1.03, -0.02)   Omnibus *P*-value for overall group difference (Intervention) = .136   1. Active Office - A and Office Ergonomics – C: 1.15 (95% CI: 0.02, 2.27), *P* = .047 2. Active Office – A and Physical Activity - B: 0.93 (95% CI: -0.47, 2.33), *P* = .189   Omnibus *P*-value for overall group difference (Organisation) = .032   1. Organisation 1 and Organisation 2: -0.27 (95% CI: -1.39, 0.85), *P* = .630 2. Organisation 1 and Organisation 3: -1.21 (95% CI: -2.26, -0.17), *P* = .024   **Break Rate (Breaks/Sedentary Hour):**  **Effect of the Different Interventions:**   1. Active Office – A: 0.85 (95% CI: -0.33, 2.02) 2. Office Ergonomics – C: 0.97 (95% CI: 0.24, 1.69) 3. Physical Activity – B: 0.02 (95% CI: -1.14, 1.18)   **Effect of the Different Organisations:**   1. Organisation 1: 1.75 (95% CI: 0.72, 2.78) 2. Organisation 2: 0.45 (95% CI: -0.51, 1.42) 3. Organisation 3: -0.01 (95% CI: -0.86, 0.84)   Omnibus *P*-value for overall group difference (Intervention) = .382   1. Active Office - A and Office Ergonomics – C: -0.12 (95% CI: -1.57, 1.33), *P* = .871 2. Active Office – A and Physical Activity - B: 0.83 (95% CI: -0.95, 2.61), *P* = .355   Omnibus *P*-value for overall group difference (Organisation) = .058   1. Organisation 1 and Organisation 2: -1.30 (95% CI: -2.82, 0.22), *P* = .094 2. Organisation 1 and Organisation 3: -1.76 (95% CI: -3.20, -0.31), *P* = .018 |
| Tan et al [16] | 2016 | 1. Individual level:    1. Three participatory skill building workshops addressing calcium intake and PA.    2. Tailored resources.    3. Calcium intake feedback. 2. Environmental level: Distribution of resources to all employees:    1. E-mails, posters, cue cars, quizzes, exhibitions, and talks.    2. An exercise CD and a 10-minute exercise poster with instructions and illustrations were provided to each participant. 3. Workshops:    1. First Workshop: Discussed the relationship between load bearing and resistance training exercises on bone cell formation and bone modelling. Allowing researchers to communicate the key message that specific types of exercise are needed to protect and promote bone health.    2. Third Workshop: Focused on helping participants identify their barriers to increasing PA levels and developing individually tailored strategies to overcome the barriers. 4. Participants would devise different types of 5-10 minute exercise routines that required minimal room and could be carried out easily at home or at the workstation. | Follow Up 1: 4-Weeks  Follow Up 2: 6-Months  **Load Bearing Moderate-to-Vigorous Physical Activity (MVPA)**  **Follow Up 1:**   1. Mean Difference: 71.8 (95% CI: 56.2, 87.5) min/week (without adjustment for potential confounders) 2. Mean Difference 61.3 (95% CI: 60.3, 62.2) min/week (after exclusion of data outside two standard deviations) 3. Mean Difference: 55.6 (95% CI: 54.5, 56.6) min/week (after adjustment for potential confounders)   **Follow Up 2:**   1. Mean Difference: 74.2 (95% CI: 71.8, 76.5) min/week (without adjustment for potential confounders) 2. Mean Difference: 51.2 (95% CI: 49.5, 52.9) min/week (after exclusion of data outside two standard deviations) 3. Mean Difference: 50.9 (95% CI: 49.3, 52.6) min/week (after adjustment for potential confounders)   **Intervention vs. Control:**  **Comparison nil leisure time load-bearing MVPA per week (%):**  Baseline: 3.7 (95% CI: -8.1, 15.5), *P* = .51  Follow Up 1: -30.9 (95% CI: -38.3, -23.6), *P* < .0005  Follow Up 2: -10.9 (95% CI: -8.1, 15.5), *P* = .04  **Comparison ≥ 60 min of leisure time load-bearing MVPA per week (%):**  Baseline: 0.1 (95% CI: -9.9, 10.1), *P* = .99  Follow Up 1: 40.3 (95% CI: 28.6, 52.0), *P* < .0005  Follow Up 2: 33.1 (19.7, 46.5), *P* < .0005 |
| Wahlstrom et al [40] | 2019 | 1. Adaptation of the physical environment (“flex office”) 2. Physical activity promoting program: including components for environmental, organisational, group and individual factors. 3. Lecture about SB and PA for all employees. 4. Workshop with managers. 5. Three-step communication campaign focus on:    1. Interruption of prolonged sitting.    2. Importance of everyday physical activity like taking the stairs, active commuting or walking meetings.    3. The usage of treadmill workstations in the office. | Reported as Estimated Means (EM). Pairwise comparisons within groups compared to baseline and model effects for group by time interactions.  Follow Up 1: 6-Months  Follow Up 2:11-Months  Follow Up 3:18-Months   1. **Sitting (min/8 hour):**   **Group x Time Effect**: *P* = .326  **Flex Office:**  Baseline: 252 (95% CI: 230, 275)  Follow Up 1: 264 (95% CI: 241, 287)  Follow Up 2: 265 (95% CI: 242, 288)  Follow Up 3: 258 (95% CI: 234, 281)  **Cell Office:**  Baseline: 242 (95% CI: 219, 265)  Follow Up 1: 238 (95% CI: 214, 263)  Follow Up 2: 254 (95% CI: 230, 278)  Follow Up 3: 250 (95% CI: 226, 274)   1. **Standing (min/8 hour):**   **Group x Time Effect**: *P* = .131  **Flex Office:**  Baseline: 189 (95% CI: 167, 211)  Follow Up 1: 168 (95% CI: 146, 190) (*P* < .01)  Follow Up 2: 169 (95% CI: 147, 192) (*P* < .05)  Follow Up 3: 175 (95% CI: 152, 198) (*P* < .05)  **Cell Office:**  Baseline: 197 (95% CI: 175, 220)  Follow Up 1: 198 (95% CI: 175, 222)  Follow Up 2: 182 (95% CI: 158, 205) (*P* < .05)  Follow Up 3: 189 (95% CI: 166, 213)   1. **Walking (min/8 hour):**   **Group x Time Effect**: *P* = .001  **Flex Office:**  Baseline: 39 (95% CI: 35, 43)  Follow Up 1: 48 (95% CI: 42, 50) (*P* < .001)  Follow Up 2: 46 (95% CI: 42, 51) (*P* < .001)  Follow Up 3: 47 (95% CI: 44, 52) (*P* < .001)  **Cell Office:**  Baseline: 42 (95% CI: 38, 46)  Follow Up 1: 44 (95% CI: 40, 49)  Follow Up 2: 45 (95% CI: 41, 49) (*P* < .05)  Follow Up 3: 41 (95% CI: 40, 46)   1. **Number of Steps/ 8 hour:**   **Group x Time Effect**: *P* = .018  **Flex Office:**  Baseline: 3602 (95% CI: 3220, 3984)  Follow Up 1: 4570 (95% CI: 4180, 4961) (*P* < .001)  Follow Up 2: 4331 (95% CI: 3930, 4731) (*P* < .001)  Follow Up 3: 4346 (95% CI: 3934, 4758) (*P* < .001)  **Cell Office:**  Baseline: 3757 (95% CI: 3365, 4149)  Follow Up 1: 4178 (95% CI: 3572, 4604) (*P* < .05)  Follow Up 2: 4300 (95% CI: 3879, 4604) (*P* < .001)  Follow Up 3: 3873 (95% CI: 3448, 4297)   1. **Time in Prolonged Sitting (min/8 hour):**   **Group x Time Effect**: *P* = .411  **Flex Office:**  Baseline: 110 (95% CI: 93, 126)  Follow Up 1: 111 (95% CI: 94, 128)  Follow Up 2: 116 (95% CI: 99, 134)  Follow Up 3: 110 (95% CI: 90, 126)  **Cell Office:**  Baseline: 99 (95% CI: 82, 116)  Follow Up 1: 93 (95% CI: 75, 112)  Follow Up 2: 98 (95% CI: 80, 117)  Follow Up 3: 106 (95% CI: 88, 125)   1. **Mean Sitting Duration (mins):**   **Group x Time Effect**: *P* = .350  **Flex Office:**  Baseline: 8.1 (95% CI: 3.2, 13.0)  Follow Up 1: 10.0 (95% CI: 4.9, 15.1)  Follow Up 2: 9.5 (95% CI: 4.1, 14.9)  Follow Up 3: 9.6 (95% CI: 3.8, 13.0)  **Cell Office:**  Baseline: 10.5 (95% CI: 5.3, 15.6)  Follow Up 1: 13.0 (95% CI: 7.0, 19.0)  Follow Up 2: 13.1 (95% CI: 7.3, 18.9)  Follow Up 3: 20.4 (95% CI: 14.5, 26.3) (*P* < .05)   1. **Number of Breaks/Sitting Hour:**   **Group x Time Effect**: *P* = .001  **Flex Office:**  Baseline: 6.7 (95% CI: 5.7, 7.7)  Follow Up 1: 6.8 (95% CI: 5.7, 7.8)  Follow Up 2: 6.5 (95% CI: 5.4, 7.5)  Follow Up 3: 7.3 (95% CI: 6.3, 8.4)  **Cell Office:**  Baseline: 7.9 (95% CI: 6.9, 9.0)  Follow Up 1: 7.3 (95% CI: 6.2, 8.4)  Follow Up 2: 6.5 (95% CI: 5.4, 7.6) (*P* < .001)  Follow Up 3: 6.5 (95% CI: 5.4, 7.5) (*P* < .001)   1. **Time in LIPA (min/8 hour):**   **Group x Time Effect**: *P* < .001  **Flex Office:**  Baseline: 148 (95% CI: 137, 158)  Follow Up 1: 136 (95% CI: 125, 146) (*P* < .001)  Follow Up 2: 128 (95% CI: 117, 139) (*P* < .001)  Follow Up 3: 138 (95% CI: 127, 149) (*P* < .01)  **Cell Office:**  Baseline: 157 (95% CI: 146, 168)  Follow Up 1: 147 (95% CI: 135, 158) (*P* < .01)  Follow Up 2: 155 (95% CI: 144, 167)  Follow Up 3: 151 (95% CI: 151, 163)   1. **Time in MVPA (min/8 hour):**   **Group x Time Effect**: *P* < .001  **Flex Office:**  Baseline: 19 (95% CI: 15, 22)  Follow Up 1: 22 (95% CI: 19, 25) (*P* < .05)  Follow Up 2: 22 (95% CI: 19, 25) (*P* < .05)  Follow Up 3: 27 (95% CI: 23, 30) (*P* < .001)  **Cell Office:**  Baseline: 16 (95% CI: 13, 19)  Follow Up 1: 18 (95% CI: 14, 21)  Follow Up 2: 22 (95% CI: 19, 26) (*P* < .001)  Follow Up 3: 19 (95% CI: 15, 22) (*P* < .05)   1. **Time in MVPA Bouts (min/8 hour):**   **Group x Time Effect**: *P* = .930  **Flex Office:**  Baseline: 5.4 (95% CI: 2.8, 8.0)  Follow Up 1: 6.6 (95% CI: 3.9, 9.2)  Follow Up 2: 6.4 (95% CI: 3.6, 9.1)  Follow Up 3: 8.8 (95% CI: 5.9, 11.6)  **Cell Office:**  Baseline: 3.9 (95% CI: 1.2, 6.6)  Follow Up 1: 5.5 (95% CI: 2.6, 8.4)  Follow Up 2: 7.5 (95% CI: 4.6, 10.4) (*P* <.01)  Follow Up 3: 5.6 (95% CI: 2.7, 8.5) |

Abbreviations: PA: physical activity; SB: sedentary behaviour; IP: intervention protocol; LIPA: light intensity physical activity; MVPA: moderate-to-vigorous physical activity; EM: estimated means; SD: standard deviation; CI: confidence intervals.
